# Biochemical Deconstruction and Reconstruction of Nuclear Matrix Reveals the Layers of Nuclear Organization

**DOI:** 10.1016/j.mcpro.2023.100671

**Published:** 2023-10-19

**Authors:** Ashish Bihani, Akshay K. Avvaru, Rakesh K. Mishra

**Affiliations:** 1 CSIR – Centre for Cellular and Molecular Biology (CCMB), Hyderabad, India; 2 Academy of Scientific and Innovative Research (AcSIR), Ghaziabad, India; 3 Tata Institute for Genetics and Society (TIGS), Bengaluru, India

**Keywords:** nuclear architecture, nuclear matrix, NuMat, nuclear organization, nucleus, nuclear skeleton, *in situ* NuMat

## Abstract

Nuclear matrix (NuMat) is the fraction of the eukaryotic nucleus insoluble to detergents and high-salt extractions that manifests as a pan-nuclear fiber-granule network. NuMat consists of ribonucleoprotein complexes, members of crucial nuclear functional modules, and DNA fragments. Although NuMat captures the organization of nonchromatin nuclear space, very little is known about components organization within NuMat. To understand the organization of NuMat components, we subfractionated it with increasing concentrations of the chaotrope guanidinium hydrochloride (GdnHCl) and analyzed the proteomic makeup of the fractions. We observe that the solubilization of proteins at different concentrations of GdnHCl is finite and independent of the broad biophysical properties of the protein sequences. Looking at the extraction pattern of the nuclear envelope and nuclear pore complex, we surmise that this fractionation represents easily solubilized/loosely bound and difficultly solubilized/tightly bound components of NuMat. Microscopic analyses of the localization of key NuMat proteins across sequential GdnHCl extractions of *in situ* NuMat further elaborate on the divergent extraction patterns. Furthermore, we solubilized NuMat in 8M GdnHCl and upon removal of GdnHCl through dialysis, *en masse* renaturation leads to RNA-dependent self-assembly of fibrous structures. The major proteome component of the self-assembled fibers comes from the difficultly solubilized, tightly bound component. This fractionation of the NuMat reveals different organizational levels within it which may reflect the structural and functional organization of nuclear architecture.

## Highlights

• Nuclear matrix (NuMat) is the insoluble nonchromatin fraction of cell nuclei.
• We subfractionate NuMat proteins and provide their relative solubility profile.
• We use *in situ* NuMat preparation to visualize the extraction patterns.
• NuMat proteins self-assemble in an RNA-dependent manner upon renaturation.
• This approach helps to look at the organization of the insoluble cellular component.

## Graphical Abstract

**Figure:**
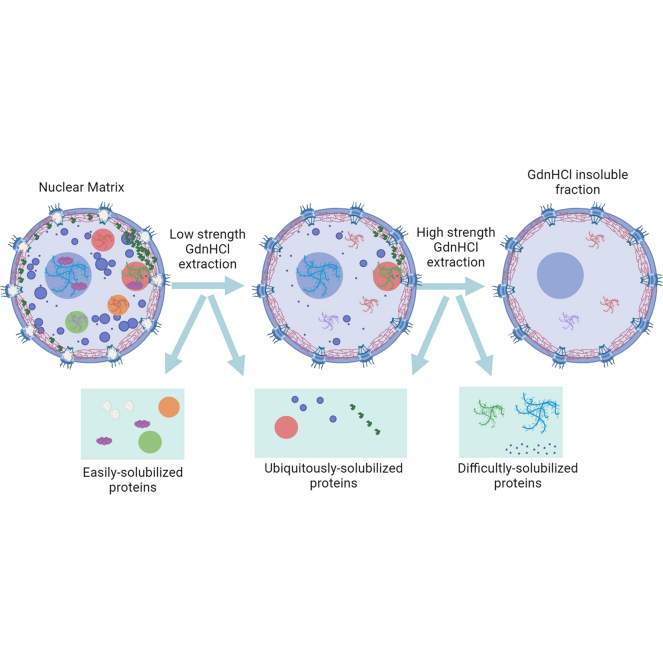

## In Brief

Much of the significant advances in cell and molecular biology rely on the soluble fraction of the cell to understand intracellular dynamics and organization. However, subcellular compartmentalization and function are heavily influenced by insoluble proteins. Self-organization through heterotypic or homotypic self-association is one of the mechanisms coming to the fore in this regard. We dissect the organization of insoluble proteins in nonchromatin nuclear space using incremental solubilization and examine the self-association tendencies of such proteins at a system-wide level.

While the advents in gene editing and high-throughput identification technologies have rapidly opened up the understanding of the functional organization of the cell, much of our current understanding of cellular organization and function is based on the soluble component of the cellular biomass which lends itself well to further biochemical and biophysical characterization (1, 2). The properties of the biomaterial insoluble to “physiological buffers” are seen through the lens of disease and aggregation. However, a large fraction of such proteins is not prone to malfunction in living cells and displays very interesting dynamic properties (3, 4). It is crucial to develop techniques and lines of thought that deal with the insoluble component of the cell to be able to understand its hidden dynamics. That said, biochemical investigations of cellular architecture through fractionation had indeed led various researchers to analyze the insoluble biomass of the cell (5, 6, 7, 8, 9, 10, 11, 12). We focus on the analysis of the insoluble component of the nucleus.

The eukaryotic nucleus is a crowded milieu that accommodates, organizes, and regulates the genome. To understand how the genome is managed, it is necessary to understand the architecture surrounding it. Biochemical investigations into the nuclear ultrastructure with different clearing buffers have yielded different residual structures, allowing exploration of different aspects of the nuclear interior (reviewed in (13)). Interestingly, the extraction of nuclei with 0.4 M NaCl and 1.4 M NaCl leads to the solubilization of the chromatin. This leaves behind a granular-fibrous insoluble structure, stipulated at the time, to be made up of precursors of ribosomal subunits (14, 15, 16). Treatment of such preparations with DNases solubilizes more material and makes the underlying insoluble material better visible, albeit highly compacted (17). Through electron microscopy, this fiber network was shown to be the skeleton of the nucleus and was termed the nuclear matrix (18, 19, 20), shortened as NuMat (21). NuMat is essentially the residual fraction after DNase I treatment and salt-detergent wash of nuclei. It contains thousands of proteins (21, 22, 23), some of them complexing various kinds of nuclear RNAs (24, 25, 26), and fragments of DNA that serve as attachment points for the chromatin to the matrix (27). Many crucial functions of the nucleus, such as transcription, replication, splicing, DNA repair, and so on, have been shown to use NuMat as a substrate (28, 29, 30, 31, 32). A huge fraction of these components is also retained in an analogous insoluble component derived from mitotic chromosomes, termed mitotic chromosomal scaffold, and is transmitted to the daughter cells (33, 34).

Despite the extensive biochemical characterization, proteomics, RNA/DNA sequencing, and microscopic studies of NuMat, we do not know the organization of these components within the NuMat fibers, or the mechanism of their assembly (35). We consider three possibilities of NuMat organization: (I) NuMat is composed of different kinds of fibers, for example, motor protein fibers (actin-myosin, tubulin-kinesin-dynein), intermediate filaments (lamin, spectrin), ribonucleoprotein (RNP) fibers (nucleolar proteins, ribosomal subunits, and heterogeneous nuclear ribonucleoproteins) (36, 37), supporting architectural proteins (megator, topoisomerase II, cohesins, condensins, and NuMat), and so on; (II) the nucleus is divided into membrane-less compartments without a uniform underlying network based solely on liquid-liquid phase separation relationships of different protein–RNA complexes. The proteins may stick to their neighbors due to a sudden change in the environment as we remove the chromatin and other buffering agents during NuMat preparation and form fibrous aggregates that are visible to us in the form of NuMat; (III) the fibers are aggregates of interchromatin RNPs forming channels for the transport of RNAs—which would form a random aggregate (38). The field of nuclear matrix has been inundated with the debate of whether such a structure exists in living cells or not (35, 39). Regardless, the biochemical dissection of NuMat fibers contains spatial information, either in the structural core and the sticky-functional fraction forming the interface with the chromatin, or in an ordered aggregate reflecting the spatial arrangement of biomolecules in subnuclear compartments.

The overall cellular biomass is dominated by proteins, and protein content is also very prominent in the insoluble biomass (23). A large number of such proteins mainly reside in the nucleus (12) and influence the organization of chromatin (40). To dissect the organization of NuMat proteins, we use a well-known chaotrope, guanidinium hydrochloride (GdnHCl), which interferes with both electrostatic and hydrophobic interactions of protein folds by forming solvation stacks (41, 42), and hence, causes proteins to denature and solubilize better than most denaturants. We reasoned that GdnHCl would readily dissolve aggregates and loosely bound proteins, leaving behind the structural elements. We have performed a sequential extraction with increasing concentrations of GdnHCl, where the gross morphology of nuclei remains unperturbed. A fraction of NuMat protein content is solubilized in a finite time for each concentration of GdnHCl. Through proteomics of different GdnHCl fractions, we observe that NuMat proteins are differentially extracted irrespective of their biophysical properties. We also observe that the difficultly solubilized proteins have a propensity for RNA-dependent reassembly. Based on these observations, we posit that insoluble preparations like NuMat contain hierarchical structures brought together by ultrastructural self-assembly in living cells. Our gradual solubilization and renaturation approaches can unlock information about the organization and function of the cell by analyzing the subfractions of such insoluble preparations.

## Experimental Procedures

All animals used in this study are *Drosophila melanogaster* used according to the procedure approved by the internal bio-safety and animal ethics committee of CSIR-CCMB.

### Preparation of NuMat from *D. melanogaster* Embryos

The protocol for the preparation of NuMat has previously been described in (23) and used here with minor modifications, described as follows.

**Supplementary Figure S1:**
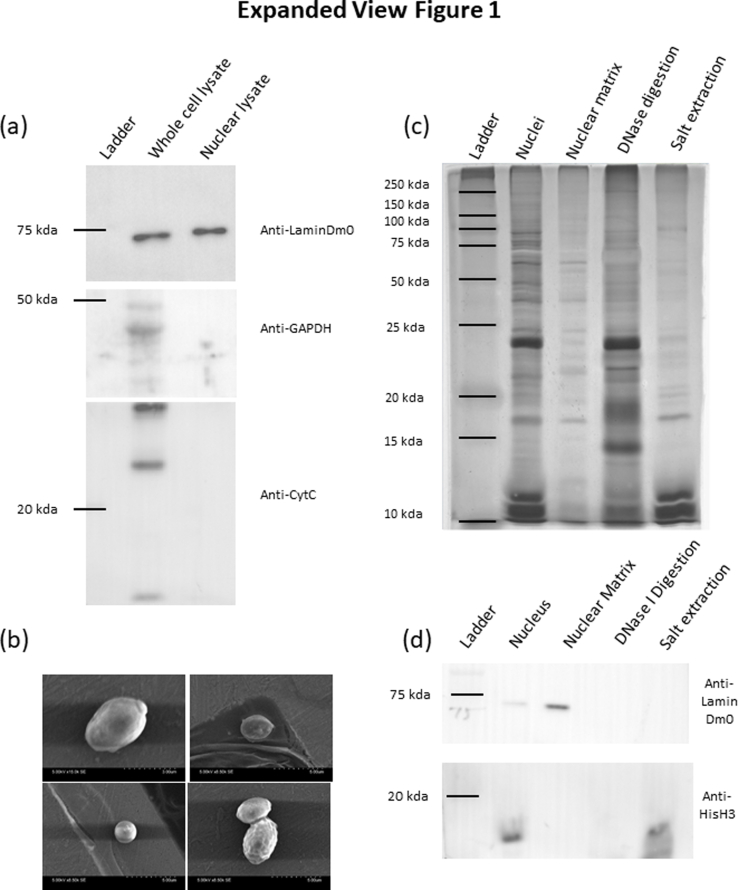
**Quality checks of NuMat preparation.***A*, the nuclear lysate was run alongside whole cell lysate and probed for Lamin Dm0, GAPDH, and Cytochrome C. While both lanes have the same amount of lamin, the nuclear lysate does not have any GAPDH or Cytochrome C (*B*) Scanning electron microscope images of the isolated nuclei; there are no cytoplasmic attachments on the outer surface, scale bars in the images. NuMat aliquot alongside nuclear lysate, DNAse I digestion supernatant, and salt extraction supernatant shown in (*A*) Silver stain of the gel (*B*) western blot probed for Lamin Dm0 and Histone H3. We see that histones are extracted in salt fraction while the nuclear matrix retains higher molecular weight proteins like Lamin.

Embryos from Canton S flies were collected overnight (16 h), and dechorionated in 50% bleach (*i.e.*, 2% sodium hypochlorite). The dechorionated embryos were lysed in freshly prepared nuclear isolation buffer (NIB) I (NIB I: 10 mM Tris-Cl pH 7.4, 40 mM KCl, 1 mM EDTA, 0.1 mM EGTA, 0.25 mM spermidine, 0.1 mM spermine, 0.1 mM PMSF, 0.25 M sucrose) with Potter-Elvehjem-type tissue grinders (six downstrokes and six upstrokes). The homogenized lysate was filtered with Miracloth of pore size 22 to 25 μm and the filtered lysate was spun at 600*g* at 4 °C for 1 min to remove debris. The crude nuclear suspension supernatant thus obtained was layered over an equal volume of NIB II (NIB II: 10 mM Tris-Cl pH 7.4, 40 mM KCl, 1 mM EDTA, 0.1 mM EGTA, 0.25 mM spermidine, 0.1 mM spermine, 0.1 mM PMSF, and 1.8 M sucrose) in an Oak Ridge style tube and spun at 6000*g* (4 °C, 10 min). The pellet containing nuclei was washed thrice with NIB I at 1000*g*, 4 °C for 10 min. The quality check for nuclear purity was done by microscopy (4′,6-diamidino-2-phenylindole (DAPI) staining, and differential interference contrast) during the experiment and Western blot (probed for Lamin Dm0 and HisH3) afterward; images of Western blot are shown in Supplemental Fig. S1*A*. Scanning electron microscopy images of purified nuclei are shown in Supplemental Fig. S1*B*.

Purified nuclei were stabilized in NIB I at 37 °C for 20 min. At this step, we also lysed 2 μl of the nuclear suspension in 0.5% SDS and measured optical density units (ODU) at 260 nm to quantify nuclei in the suspension by quantifying total DNA. The stabilized nuclei were pelleted (at 1000*g*, 4 °C for 10 min) and resuspended in DNase I digestion buffer (20 mM Tris-Cl pH 7.4, 20 mM KCl, 70 mM NaCl, 10 mM MgCl_2_, 0.5% Triton X-100, μg/ml DNase I, 0.1 mM PMSF; DNase I added after resuspension). The suspension was incubated at 4 °C for 1 h. Nuclei were pelleted at 1000*g* at 4 °C for 10 min. The pellet was resuspended in extraction buffer I (5 mM Hepes pH 7.4, 2 mM KCl, 2 mM EDTA, 0.25 mM spermidine, 0.1 mM spermine, 0.1 mM PMSF, 0.5% Triton X-100, and 0.4 M NaCl) and with extraction buffer II (5 mM Hepes pH 7.4, 2 mM KCl, 2 mM EDTA, 0.25 mM spermidine, 0.1 mM spermine, 0.1 mM PMSF, 0.5% Triton X-100, 2 M NaCl) sequentially and rotated (10 rpm, room temperature (RT) for 10 min in each case). The suspension was pelleted at 3000*g* for 15 min at RT. To keep track of quantity, NuMat preparation was resuspended in extraction buffer II (10 μl per ODU) and the total suspension was split into 500 μl or 50 ODU equivalents. These suspensions were spun down, washed twice in washing buffer (WB: 10 mM Tris-Cl pH 7.4, 20 mM KCl, 1 mM EDTA, 0.25 mM spermidine, 0.1 mM spermine, 0.1 mM PMSF) and were stored at −30 °C. A silver-stained gel profile of NuMat proteins in contrast with an equivalent amount of nuclei, DNase I digestion supernatant, and salt extraction supernatant is shown in Supplemental Fig. S1*C*. Retention of architectural protein Lamin Dm0 and removal of chromatin protein histone H3 is shown in Western blot in Supplemental Fig. S1*D*.

### Subfractionation of NuMat with Guanidinium Hydrochloride (GdnHCl)

**Fig. 1:**
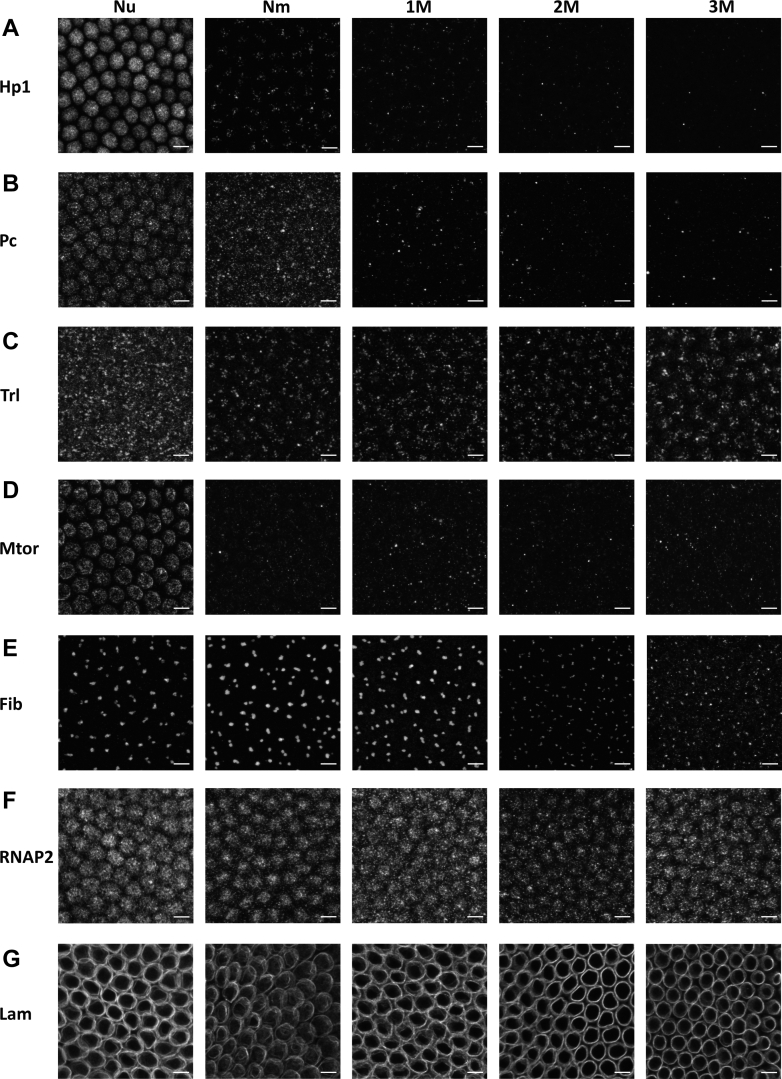
**Extraction patterns ofwell-knownNuMat proteins in*****in situ*****NuMat follow a trend similar to proteomics.** Shown here are images of *Drosophila* embryos at the 13th-15th stage (∼2 h), at100× magnification and 3× zoom (Nu for Nuclei, Nm for NuMat, and 1M, 2M, 3M extracts). Each row shows the sum projection of a sheet of nuclei immunostained for: (*A*) Hp1a (*B*) polycomb (*C*) trithorax-like (*D*) megator (*E*) fibrillarin (*F*) RNAP2 (*G*) lamin; in greyscale to assess the extent of extraction for the sequentially extracted samples. The scale bar represents 5 μm in all images. NuMat, nuclear matrix.

**Fig. 2:**
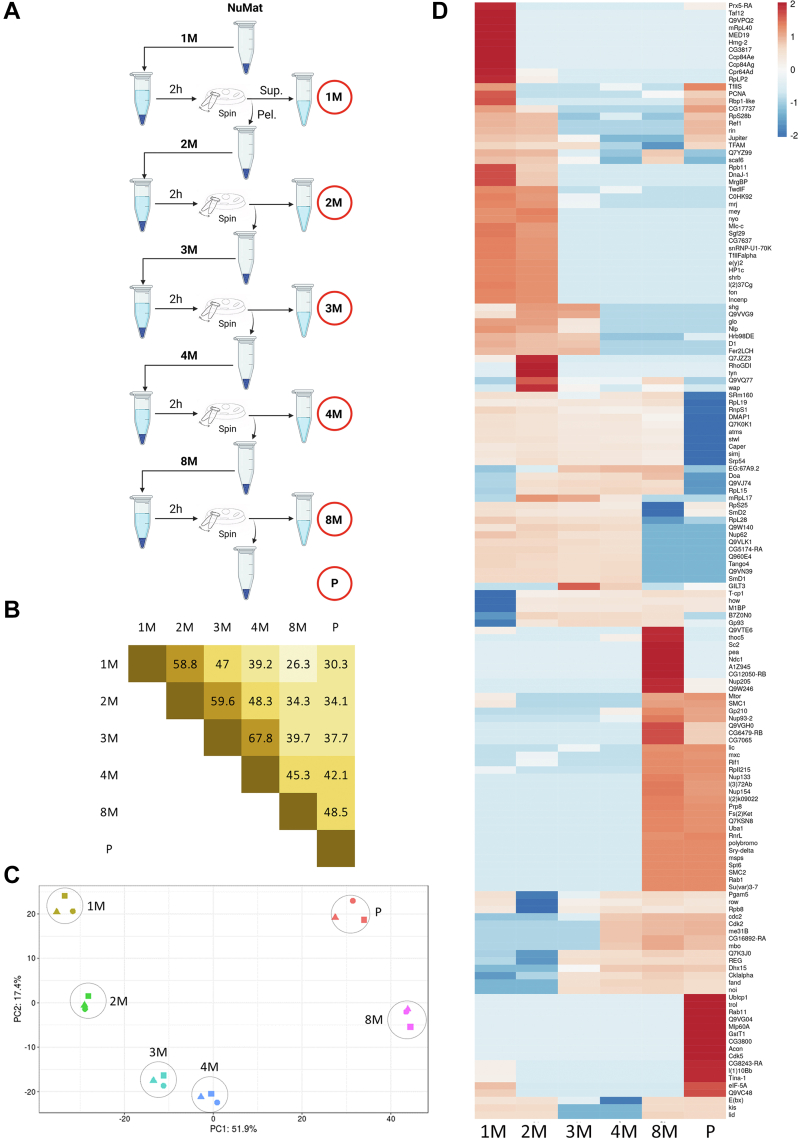
**NuMat proteins show differential enrichment in different GdnHCl extraction fractions.***A*, for sequential extraction of NuMat with different GdnHCl concentrations the procedure is as described in the schematic; NuMat is resuspended in extraction buffer of the mentioned GdnHCl concentration, incubated for 2 h at 25 °C, and then pelleted down. The supernatant is taken as an extract while the pellet is resuspended in the extraction buffer containing a higher concentration of GdnHCl, repeated till 8M. The 1M, 2M, 3M, 4M, and 8M extracts and the final pellet (P) are subjected to proteomics (marked with *red circles*). *B*, pairwise percentage overlap for NuMat fractions. Shown here in a 6 × 6 matrix is the overlap of each fraction with another fraction, each box corresponding to the notation of two concentrations contains the percentage overlap of protein lists for those two fractions. The higher the extent of overlap, the more intense the color of the box. We see that the extraction profiles of fractions closer in terms of GdnHCl concentration overlap better. *C*, replicate correlation for all replicates across all fractions in the sequential extraction of *Drosophila* embryo NuMat and with different concentrations of GdnHCl. Each dot represents one sample (one replicate of one fraction, as labeled). The separation between dots denotes the difference between them while dots clustering together have a similar profile. Replicates of each fraction cluster together well and are distinct from other fractions. *D*, quantitative enrichment profile of proteins across the fractions of NuMat. LFQ intensities of GdnHCl fractions of NuMat were compared using LFQ-analyst, filtered by fold change (>25log_2_-fold, *p* < 0.001) without imputation and Benjamini-Hochberg FDR correction. Fraction-wise relative enrichment scores of selected proteins were calculated using DEP and plotted in a heatmap using ClustVis. Columns represent fractions (1M, 2M, 3M, 4M, 8M, and P) and *rows* represent individual proteins. The *red color* shows the enrichment of proteins and the *blue color* shows their depletion. We see a huge number of proteins are highly differentially enriched in different fractions and there is a stark division between higher-concentration and lower-concentration extractions. DEP, Differential Enrichment analysis of Proteomics data; FDR, false discovery rate; GdnHCl, guanidinium hydrochloride; LFQ, label-free quantitation; NuMat, nuclear matrix.

**Supplementary Figure S2:**
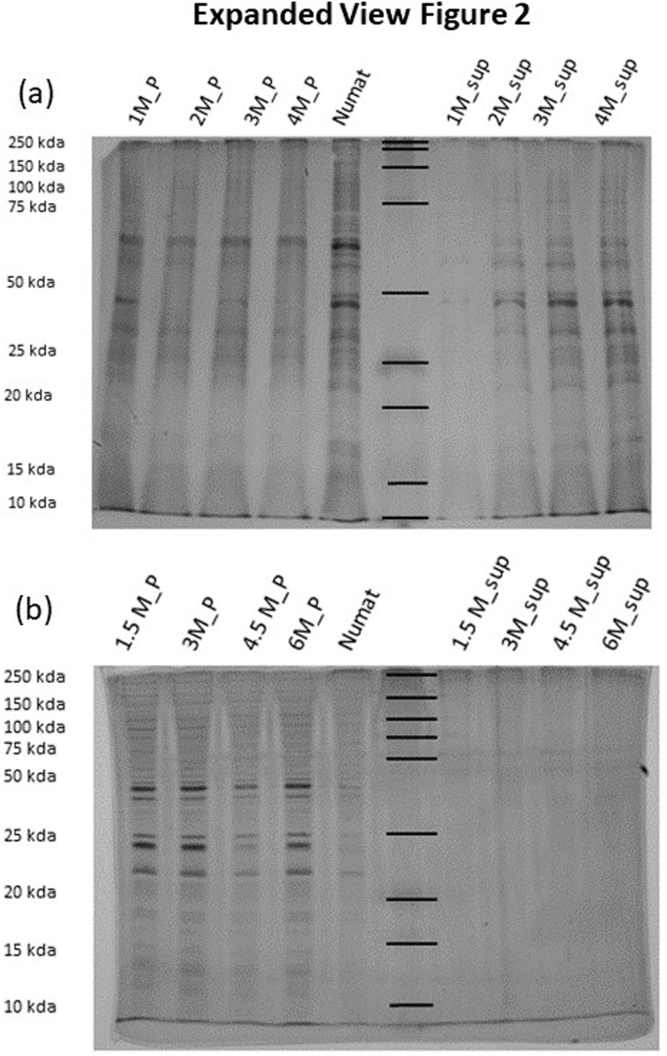
**Protein profile of the NuMat extraction with GdnHCl and Urea.** Four separate NuMat aliquots were resuspended in 1M, 2M, 3M, and 4M GdnHCl solution, and another four in 1.5M, 3M, 4.5M, and 6M Urea. Supernatants were loaded in lanes labelled XM_Sup. Pellets were loaded in lanes labelled as XM_P. Large-scale solubilization is visible in GdnHCl supernatants (*A*) and none in Urea supernatants (*B*). There is a marked difference between the proteins solubilized by GdnHCl and the proteins left in the pellet.

**Supplementary Figure S3:**
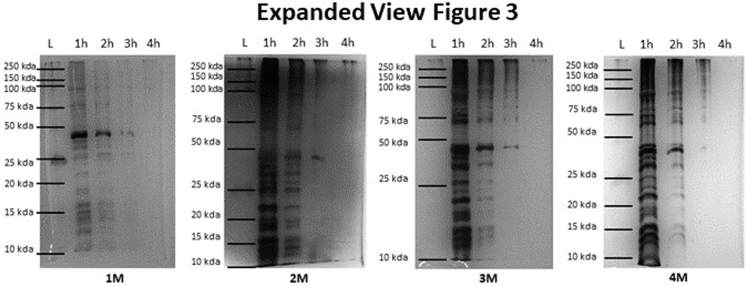
**Check for leeching in NuMat extraction with GdnHCl by sequential extraction with the same concentration.** Four separate NuMat aliquots were resuspended in 1M, 2M, 3M, and 4M GdnHCl solution. The suspension was pelleted every hour and the pellet was resuspended in a fresh solution of the same GdnHCl concentration. Here shown are the silver stains of the 1-hour protein extraction profile for each concentration. Maximum extraction happens within 2 h in each case.

**Supplementary Figure S4:**
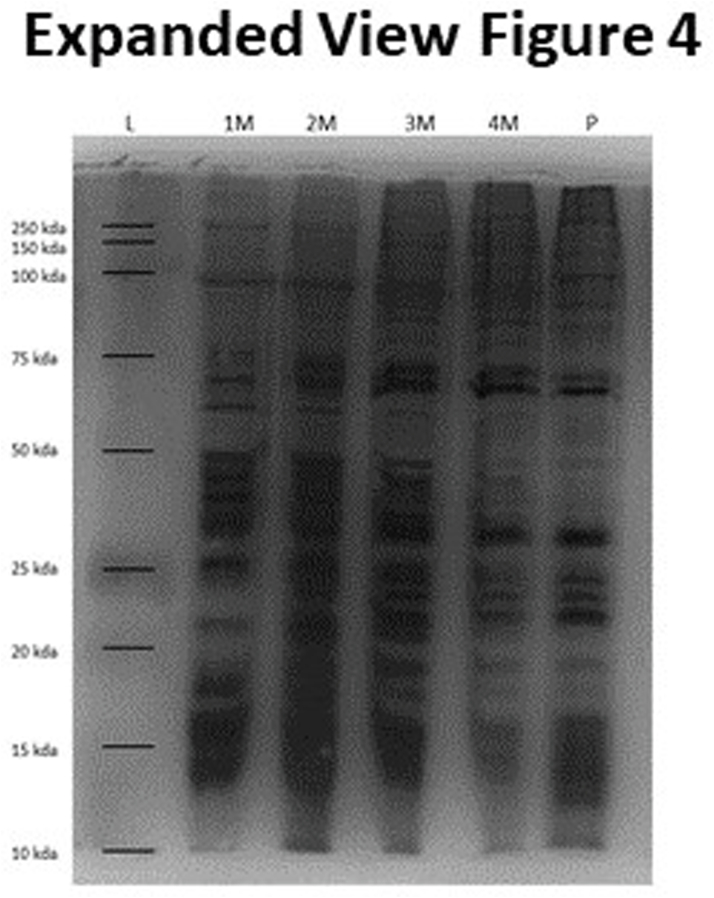
**NuMat extract profiles visualized on SDS-PAGE.** NuMat aliquot was sequentially extracted with 1M, 2M, 3M, and 4M GdnHCl solutions according to the experimental schematic shown in Fig 2*A*. All four supernatants and the pellet were loaded. The profiles are slightly different.

For both isolated nuclei and intact embryos (Figs. 1 and 2), NuMat was subfractionated by sequential extraction with GdnHCl extraction buffers (1M, 2M, 3M, 4M, and 8M) as per the experimental schematic shown in Figure 2*A*. The experimental conditions mentioned in Figure 2*A* were finalized through the standardization process described in Supplemental File S1, and results shown in supplemental Figs. S2–S4.

The NuMat aliquot (derived from 50 ODU nuclei) was resuspended in 200 μl 1M GdnHCl (in 20 mM Mops, pH 7.4) and extracted for 2 h at RT, and pelleted down (5000*g* at RT for 5 min). The supernatant was removed and saved for proteomics at −30 °C. The pellet from the extraction was resuspended in 200 μl GdnHCl buffer of the next concentration for the next extraction. The sequential extraction process subfractionates the NuMat preparation in six parts: 1M extract, 2M extract, 3M extract, 4M extract, 8M extract, and pellet—encircled in red in Figure 2*A*. (Fig. 2*A*, and the graphical abstract were made using Biorender)

### SDS-PAGE of GdnHCl Fractions

To ensure a good SDS-PAGE run and proper size resolution, all the samples loaded on SDS-PAGE have to have equal GdnHCl concentrations. For this purpose, 80 μl extracts from each GdnHCl fraction (20 ODU equivalent) were taken in separate tubes. A 20 μl mixture of 8M GdnHCl and Milli-Q water prepared in different proportions for individual fractions was added to the respective extracts to adjust the concentration to 3M. The 100 μl extracts thus obtained were boiled with 25 μl 5× Laemmli buffer and loaded on the gel. The gel was run at 80 V till the time the GdnHCl aggregate formed in the wells and the dye front entered the stacking gel. At this point, the wells were flushed and the buffer in the gel compartment was changed. After this, the gel was run like a regular SDS-PAGE gel.

### Renaturation

**Fig. 3:**
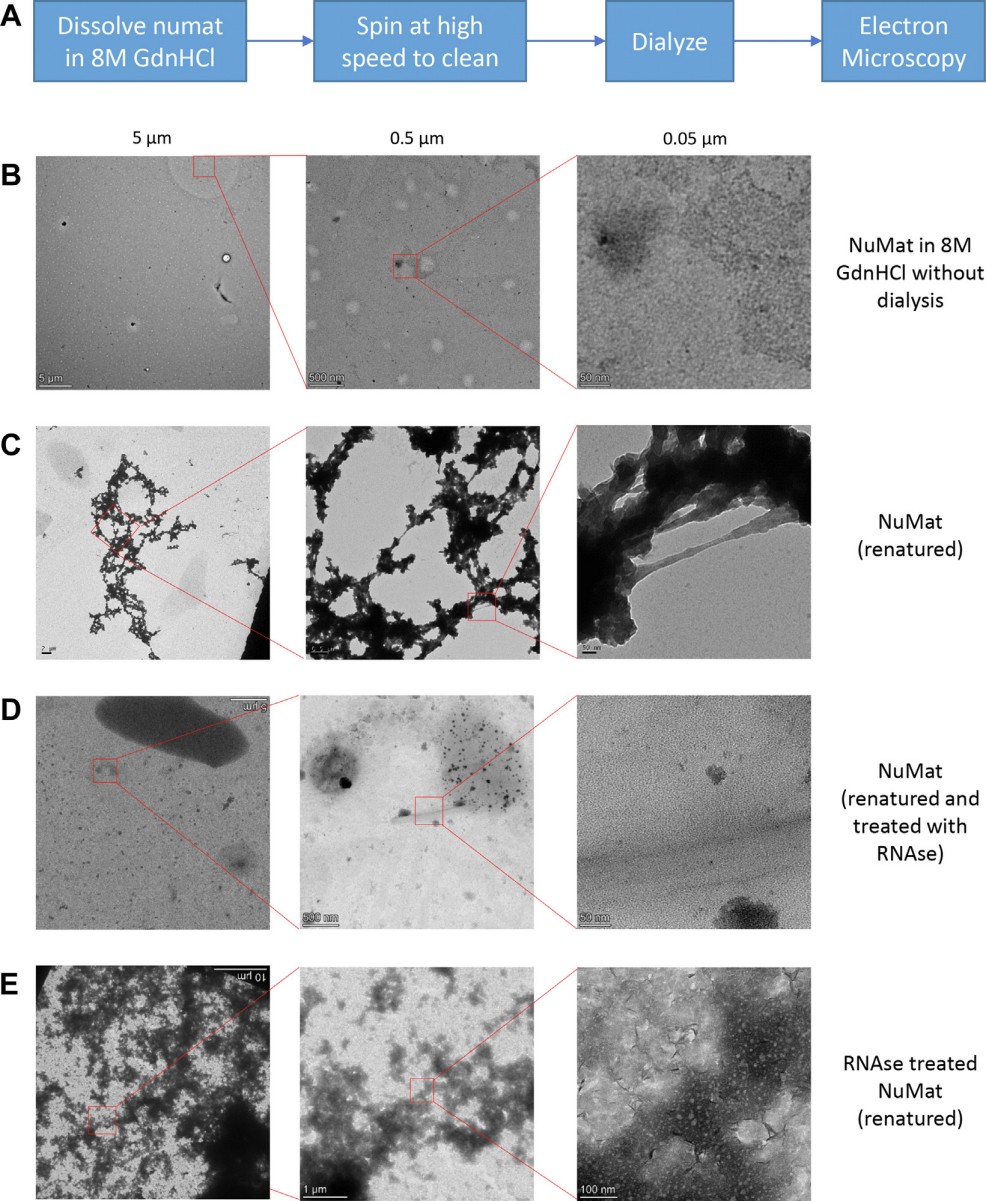
**NuMat proteinsself-assemblein a network of fibers.** NuMat proteins were dissolved in 8M GdnHCl and any debris was removed by high-speed spin, schematic shown in (*A*). The lack of ultrastructure in the 8M denaturant is shown in (*B*). This solution, when dialyzed to remove GdnHCl, forms networks of fibers (shown in (*C*)). When the fibers obtained in (*C*) were treated with RNAse, the fibers collapsed (shown in (*D*)). Similarly, NuMat preparation with RNAse treatment did not yield a contiguous fiber upon renaturation (shown in (*E*)). For all experiments, pictures were taken at three different magnifications (scales of 5 μm, 0.5 μm, and 0.05 μm unless specified). The magnified area is outlined in a *red square*. GdnHCl, guanidinium hydrochloride; NuMat, nuclear matrix.

**Fig. 4:**
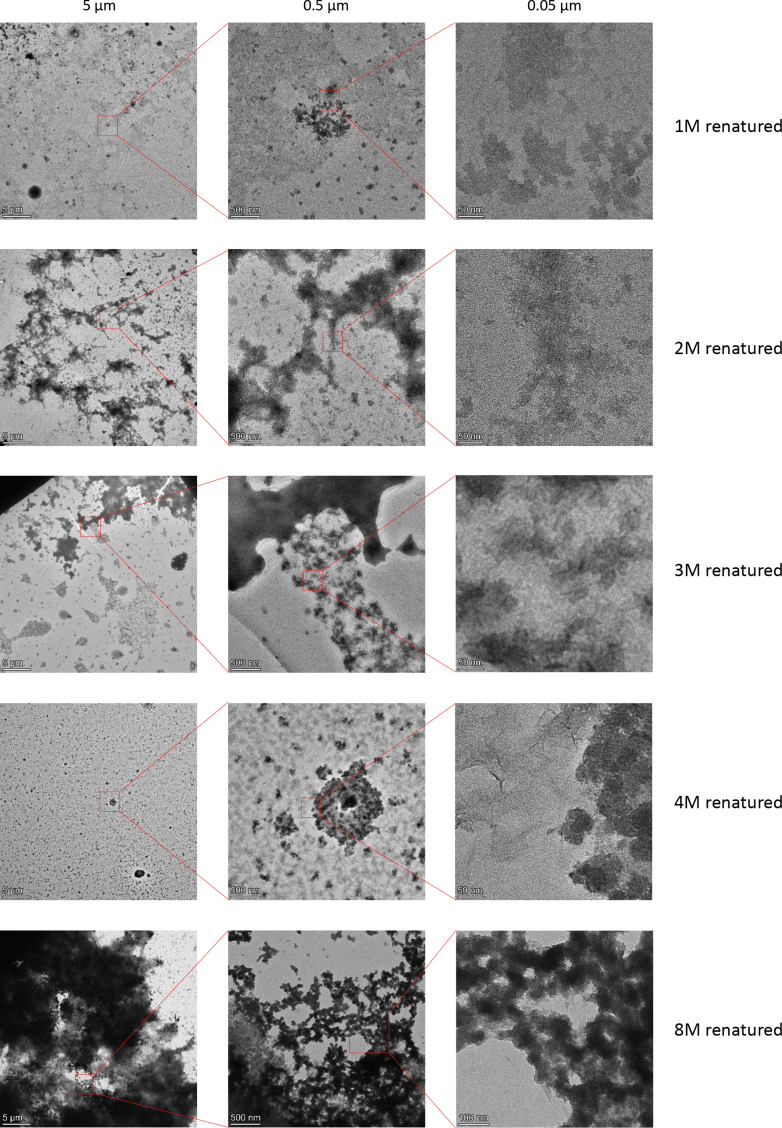
**Difficultly solubilized proteins contribute the most toself-assemblingfibers.** The extraction fractions of NuMat prepared from sequential extraction by GdnHCl (schematic in Fig. 2*A*) were renatured (schematic in Fig. 3*A*), spun, resuspended in 10 μl water and spotted on the grid. Each *row* shows electron micrographs for each extraction fraction renaturate (1M renatured, 2M renatured, 3M renatured, 4M renatured, and 8M renatured, as labeled) at three different magnifications (at scales 5 μm, 0.5 μm, and 0.05 μm, marked on top of each column). The area magnified from one magnification to the next is shown using a *red box*. GdnHCl, guanidinium hydrochloride; NuMat, nuclear matrix.

The experimental schematic for renaturation is shown in Figure 3*A*. The NuMat aliquot or the other preparations as specified in Figures 3 and 4 were dissolved in 200 μl 8M GdnHCl by vigorous vortexing. The solution was precleared of debris by centrifugation at 21000*g* for 10 min at RT. The supernatant was dialyzed against 20 mM Mops buffer (pH 7.4) overnight. A carbon-coated copper/nickel grid was dipped in the dialyzed renaturate and shaken lightly. The liquid was removed using a blotting sheet and the grid was stained with uranyl acetate. The samples were visualized in Jeol JEM-2100 (200 kV) or Thermo Fisher Scientific Talos L120C (120 kV) transmission electron microscopes. For proteomics, the NuMat renaturate was spun down in a pellet and processed.

### Proteomics and Data Analysis

GdnHCl SDS-PAGE gels of the samples were Coomassie stained. Each lane was cut into three strips of higher, medium, and low molecular weight strips. Each strip was separately diced into 1 mm^3^ pieces which were washed three times with wash buffer (25 mM NH_4_HCO_3_ and 50% acetonitrile). The pieces were dehydrated using 100% acetonitrile and dried in a SpeedVac followed by 10 mM DTT treatment in 25 mM NH_4_HCO_3_ (45 min at 55 °C). After this, the cubes were saturated with 55 mM iodoacetamide (30 min at 37 °C). The pieces were dried again with 100% acetonitrile and subsequent SpeedVac. The dried pieces were wetted in 10 ng/μl trypsin gold (Promega) in 25 mM NH_4_HCO_3_ and incubated at 37 °C overnight (16 h). After 16 h, trypsin action was stopped and peptides were eluted with 2.5% TFA in 50% acetonitrile in two sequential washes. The supernatant from both washes was collected in fresh tubes and dried with SpeedVac. The pellet was resuspended in 0.1% TFA and 2% acetonitrile. This sample was desalted with Millipore p10 Ziptip C18. The elution buffer was evaporated using SpeedVac and the peptides were resuspended in 0.1% formic acid and 2% acetonitrile.

**Fig. 5:**
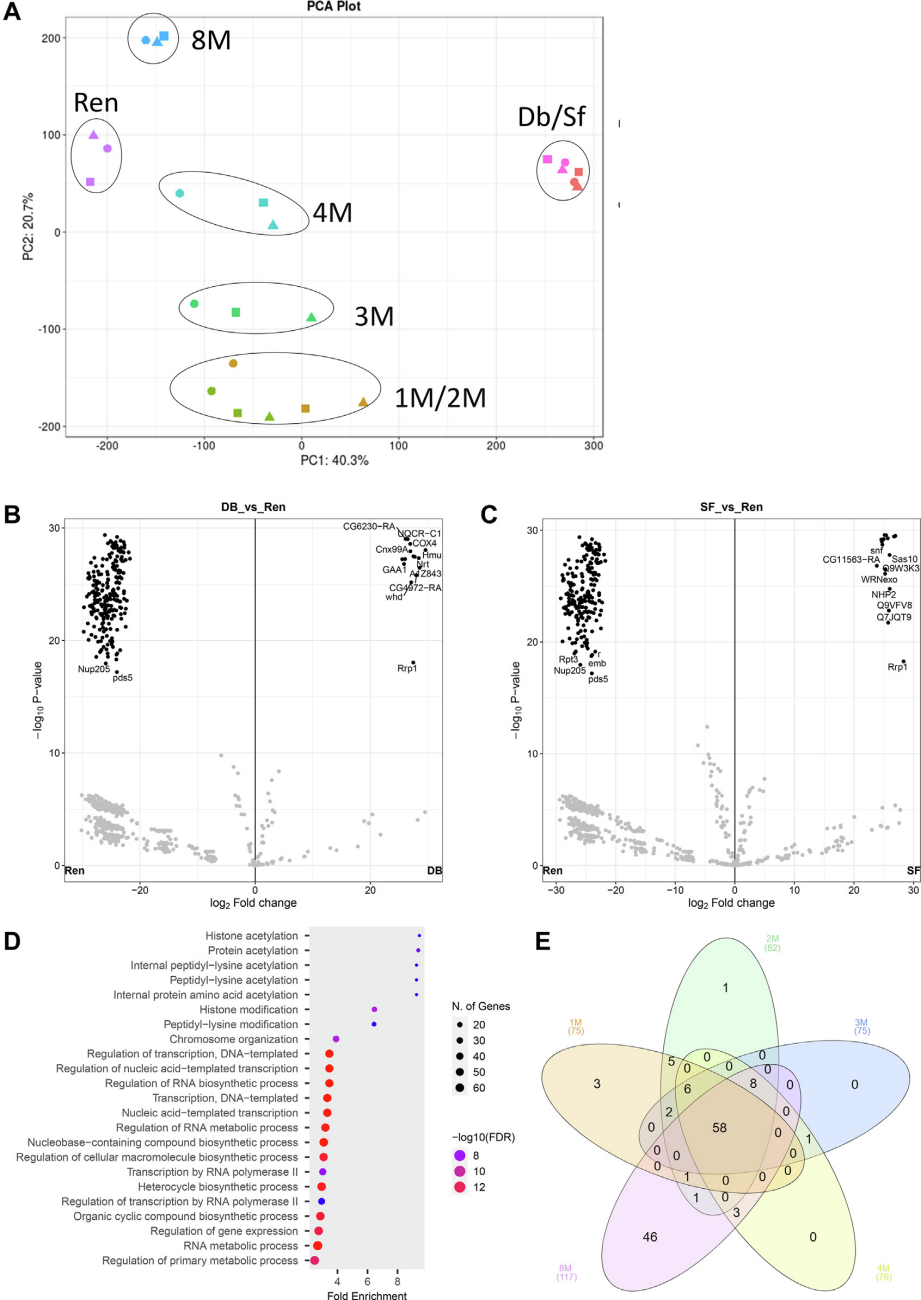
**NuMat proteins responsible for reassembly are difficultly solubilized and belong to diverse pathways.***A*, the proteome profile of renatured pellet is different from non-NuMat nuclear fractions (DNase I digestion fraction and salt extraction fraction) and similar to the 8M extraction fraction. Shown here is the PCA plot where the replicates of individual fractions cluster together. The renatured fiber-forming fraction clusters farthest away from nonfiber forming fractions (DNAse I digestion fraction and salt extraction fraction), and closer to the extraction fractions, especially 8M. *B*, quantitative comparison of the proteomic profile of renatured fraction with DNase I digestion fraction. The horizontal axis shows the log_2_ fold change of the average LFQ intensity ratio for an individual protein for DNAse I digestion (Db) *versus* renaturation (ren), and the vertical axis shows the statistical significance of the fold change in terms of –log10 of the *p*-value. We see a distinct cluster of 273 proteins that have >2^20^ fold change and <10^−10^*p*-value, denoting proteins that are principal exclusive contributors to fiber-forming renaturation fraction (highlighted in *red square*). *C*, quantitative comparison of the proteomic profile of renatured fraction with salt extraction fraction. The horizontal axis shows the log_2_ fold change of the average LFQ intensity ratio for an individual protein for salt extraction (Sf) *versus* renaturation (ren), and the vertical axis shows the statistical significance of the fold change in terms of –log10 of the *p*-value. We see a distinct cluster of 219 proteins that have >2^20^ fold change and <10^−10^*p*-value, denoting proteins that are principal exclusive contributors to fiber-forming renaturation fraction (highlighted in *red square*). *D*, gene Ontology analysis of core renaturation factors (made with ShinyGO) shows high fold change enrichment of GO terms related to biological processes like translation, histone modification, and chromosome organization. *Y*-axis shows various biological processes enriched in core renaturation factors, *X*-axis shows fold change, the dot size shows the absolute protein count for a pathway, and the dot color represents the *p*-value of enrichment (*red* means more significant). *E*, contribution of extraction fractions to core renaturation factors. The proteins from the overlap of core renaturation fraction with individual extraction fractions were used as input in a 5-way Venn diagram (made with interactiVenn). We see that the biggest contribution to CRF comes from 8M, both unique and overlapping. CRF, core renaturation fraction; LFQ, label-free quantitation; NuMat, nuclear matrix; PCA, principal component analysis.

**Supplementary Figure S5:**
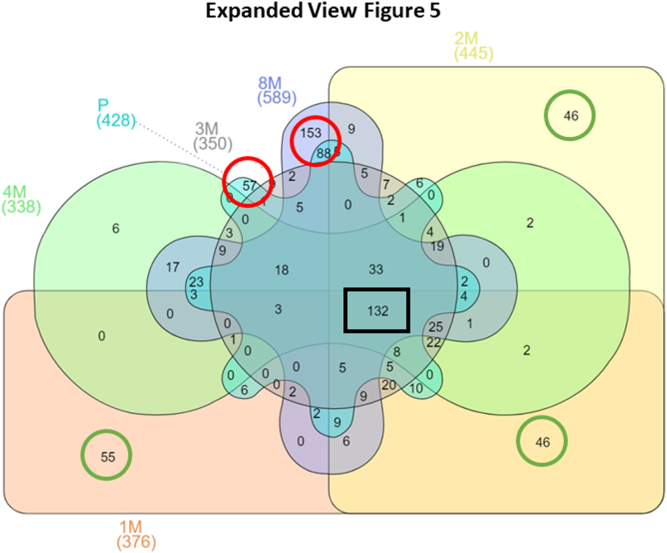
**Overlap of proteins across fractions, compared by 6-way Venn diagram: We observe that the qualitative profiles of 1M, 2M, 3M, 4M, 8M, and P fractions have 132 proteins common to all fractions (highlighted in a *black square*).** High-concentration extractions (8M and P, highlighted in *red circles*) and low-concentration extractions (1M and 2M, highlighted in *green circles*), emerge as larger unique clusters as opposed to 3M-4M. Thus, there is a clear divergence in easily-solubilized (*green*) and difficultly-solubilized (*red*) groups.

**Supplementary Figure S6:**
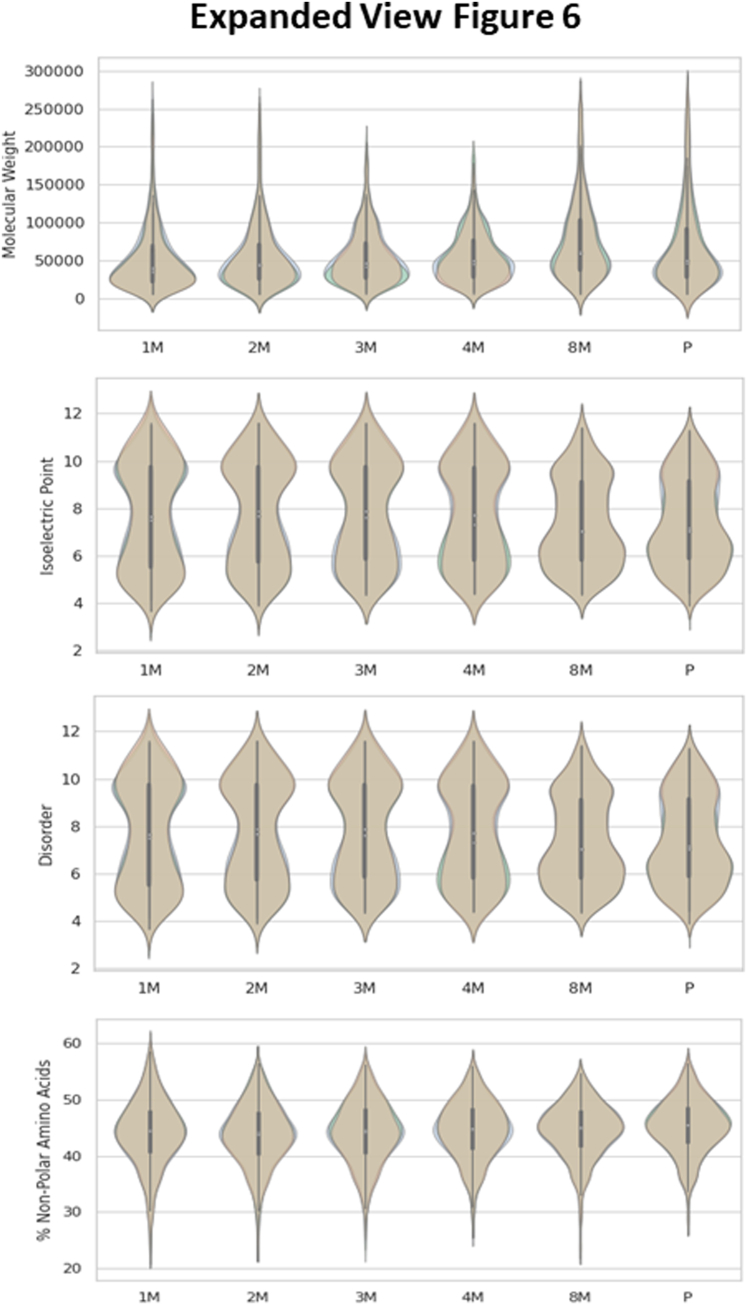
**Comparisons of broad biophysical property histograms across GdnHCl fractions.** Shown here are violin plots of protein number distribution for (*A*) Molecular weight, (*B*) Isoelectric point, (*C*) Intrinsic Disorder Percentage (data from MobiDB (48)), (*D*) Non-polar Amino Acids for all three replicates of each fraction. There is no biophysical bias in the extraction of embryo NuMat proteins at different GdnHCl concentrations.

The spectra were collected on Thermo Fisher Scientific Q-Exactive HF on a 60-min acetonitrile gradient and analyzed with MaxQuant (version 1.5.7.4) at default settings. Search parameters of this setting are provided in Supplemental Table S1. The search was conducted against the *D. melanogaster* proteome downloaded from Uniprot on February 10, 2017, with 21,994 entries. Proteins that had at least two unique peptides and nonzero label-free quantitation (LFQ) intensities across all three biological replicates were considered for the analysis. All the replicates of all the fractions were subjected to principal component analysis (PCA), considering the correlation between LFQ intensities associated with all the protein lists of individual samples (43). The differential enrichment analysis for these proteins’ LFQ intensities across different fractions was performed with Differential Enrichment analysis of Proteomics data (DEP) and LFQ-analyst (43, 44). Proteins that showed more than 25log_2_ fold LFQ intensity difference statistically significant (*p* < 0.001) were chosen. Their differential enrichment score was plotted in a heatmap using ClustVis, with average distance clustering of rows (45). InteractiVenn and ShinyGO have been used to look at the distribution and pathway enrichment of protein lists (46, 47) (Fig. 5 and supplemental Fig. S5). Biophysical properties of proteins extracted from MobiDB were plotted using R (supplemental Fig. S6) (48).

**Fig. 6:**
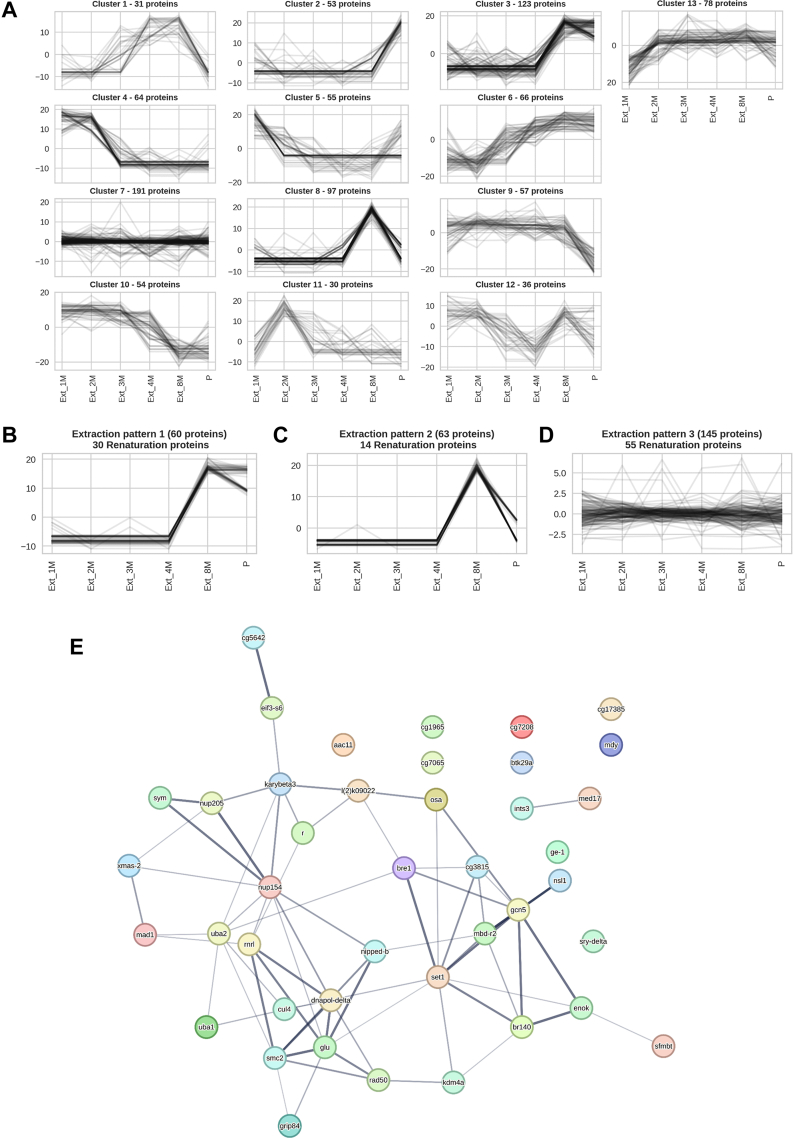
**Refined subgroups of difficultly solubilize proteins show significantly associated proteins.***A*, clusters of extraction patterns identified among NuMat proteins by k-means clustering, with *x*-axis having GdnHCl extraction fractions from 1M on the left to P on the right and *y*-axis having the DEP scores. The headers include the number of proteins present in each cluster. *B*–*D*, a refined cluster of proteins shows three prominent extraction patterns which also retain the most number of renaturing proteins (*B*). Extraction pattern 1-proteins enriched specifically in 8M fraction and the pellet with a total of 60 proteins of which 30 proteins renature (*C*). Extraction pattern 2–proteins enriched specifically to 8M fraction with a total of 63 proteins of which 14 proteins renature (*D*). Extraction pattern 3—ubiquitously extracted proteins with a total of 145 proteins of which 55 proteins renature. *E*, String-db analysis significant association of renaturing proteins found in extraction patterns 1 and 2 which represent difficultly solubilized proteins. DEP, Differential Enrichment analysis of Proteomics data; GdnHCl, guanidinium hydrochloride; NuMat, nuclear matrix.

We performed K-means clustering of the proteins based on their DEP score distributions across the extraction fractions (Fig. 6*A*). Silhouette score analysis indicated optimal clustering when k is set to 13. We refined the clusters to select the proteins which are strictly following a prominent pattern. The refinement was done by further applying k-means clustering on the proteins of individual clusters. Finally, the subclusters that show high cluster tightness and adherence to a prominent extraction pattern are analyzed for the presence of renaturing proteins. (prominent ones are shown in Fig. 6, *B*–*D*). The renaturing members of extraction pattern 1 and extraction pattern 2 (Fig. 6, *B* and *C*) were further subjected to string-db analyses (Fig. 6*E*).

### *In situ* NuMat Preparation and GdnHCl Extraction

The protocol for preparing *in situ* NuMat has previously been published (49, 50). To mimic the extraction procedure described in Methods section B, we eliminated formaldehyde crosslinking. The modified procedure and subsequent sequential extractions are described as follows.

Embryos collected from Canton S flies were washed and dechorionated in 50% bleach until dechorionated. For the removal of the vitelline membrane, the embryos were rotated with 50% heptane in 1× PBS for 10 min. The embryos at the heptane-PBS interface were taken for further processing in a heptane-methanol (1:1) mix. After this step, the embryos that sat at the bottom of the tube were washed with cold methanol. After the fixed embryos became nonsticky, they were stored in methanol at −30 °C. For NuMat preparation, embryos were rehydrated by drop-by-drop addition of 1× PBS, pH 7.4 with 0.5% Triton X-100 (PBT) and washed thrice with cold PBT (20 min each time). Next, the embryos were stabilized at 37 °C for 20 min. After this, the embryos were treated with extraction buffer I (0.4 M NaCl) and extraction buffer II (2 M NaCl), for 20 min each. The stabilized embryos were submerged in DNase I digestion buffer (30 min). The resulting *in situ* NuMat was washed thrice with cold PBT (20 min each time).

For sequential extraction of *in situ* NuMat, 1M GdnHCl solution was added to three aliquots of *in situ* NuMat and rotated at RT for 2 h—the solution was carefully aspirated at the end. One aliquot was subjected to three PBT washes, 20 min each. Meanwhile, 2M GdnHCl solution was added to the other two aliquots and rotated at RT for 2 h. One aliquot was subjected to three PBT washes, 20 min each. Meanwhile, 3M GdnHCl solution was added to the remaining aliquot and rotated at RT for 2 h. This aliquot was subjected to three PBT washes, 20 min each. The embryo structure collapses at GdnHCl concentrations higher than 3M.

### Immunostaining and Confocal Imaging of Extracted *in Situ* NuMat

The embryo derivative samples (Embryos, *in situ* NuMat, and *in situ* NuMat extracted 1M, 2M, and 3M GdnHCl buffers) were blocked by rotating in 3% bovine serum albumin in PBT for 2 h at 4 °C. After a quick rinse with cold PBT, primary antibody solution was added and the suspension was rotated overnight at 4 °C (16 h). Next, after three washes with cold PBT, the sample was rotated with the secondary antibody solution (4 °C for 3 h). The antibody solution was aspirated and DAPI was added in appropriate amounts to the sample bed volume. The sample was washed thrice with cold PBT, 20 min each time. The sample was mounted in Vectashield mounting media without DAPI and sealed.

Mounted samples were imaged with Olympus FV3000 confocal microscope at 100× magnification with 3× zoom, taking near-saturation voltage values. The raw data were processed with ImageJ (https://imagej.net/software/imagej) to get the same number of slices of all scans of one sample, to take the sum intensity projection, and to add the scale bar.

Details of important reagents and antibodies are specified in tables in Supplemental File S1.

### Experimental Design and Statistical Rationale

For each liquid chromatography with tandem mass spectrometry-based proteomics experiment, three biological replicates from independent biological preparations were analyzed. The SDS-PAGE lane of each replicate was cut into three strips based on size to improve proteomics coverage and their data were merged in the MaxQuant run by specifying the spectra from strips as fractions of the same replicate. Peptide and protein identification false discovery rates of 1% were used. Only proteins identified in all three biological replicates with two or more peptides and nonzero total LFQ intensity in at least one fraction were considered true fraction components and were used for further analysis to exclude any spurious identifications.

PCA(with LFQ-Analyst) was used to judge the reliability of quantification measurements between biological replicates. The differential enrichment of protein across GdnHCl extraction fractions was judged by the fold change difference (>25log_2_-fold) between triplicate LFQ-intensities and the associated *p*-value (<0.001) (using LFQ-analyst and DEP). For identifying the proteins involved in reassembly, we compared NuMat renaturation fraction with DNase I digestion fraction and salt extraction fraction, we have instead looked at the cluster emerging through the volcano plot and subsequent Gene Ontology (GO) analysis of the subset of this cluster, which does show very high fold change (>20log_2_-fold) and *p*-value (<0.001).

## Results

The spatial organization and compartmentalization of chromatin in the eukaryotic nuclei dictate the fate and behavior of a cell. The nonchromatin component of the nucleus forms an environment around chromatin that shapes and is shaped by the activity and organization of chromatin (51, 52). NuMat, the insoluble component of the eukaryotic nucleus, serves as a snapshot of the nonchromatin nuclear architecture (22) (Preparation shown in Supplemental Fig. S1). Therefore, we reason that a systematic examination through differential solubilization and subfractionation of NuMat would shed light on the organization of components within NuMat, yielding insights into gross nuclear architecture. However, any further subfractions of NuMat have not been characterized. The filamentous architecture of NuMat has been observed to be eliminated by proteases or sodium deoxycholate treatments (53), while treatment with RNAse causes the fibrous structures in the nuclear interior to collapse (36, 54). RNAse treatment also disrupts the overall chromatin organization (55, 56). On the other hand, various salts/detergents (22), and urea (Supplemental Fig. S2*A*) do not show any significant solubilization. A gradual biochemical extraction method is needed—which we could realize by using buffers with increasing concentrations of GdnHCl (Supplemental Fig. S2*B*).

We used 1M, 2M, 3M, and 4M concentrations of GdnHCl to test and optimize fractionation (the optimization process is described in Supplemental File S1). We show that incubation with the GdnHCl extraction buffer of a particular concentration is able to solubilize a distinct set of proteins at that concentration with no further extraction happening upon longer incubation (Supplemental Fig. S3). The finite extraction in finite time is a strong indication that NuMat fibers might not result from random clumping of nonchromatin proteins. The finite extraction at each concentration enabled us to extract NuMat preps with GdnHCl in a sequential manner (Supplemental Fig. S4). The sequential extraction of NuMat yielded six fractions, labeled with the respective GdnHCl concentration. As shown in Supplemental Fig. S4, the sequential extraction fractionation shows interesting solubilization patterns. Some proteins are solubilized by low ionic strength GdnHCl buffers (1M and 2M) while others remain insoluble even after extraction with 4M GdnHCl buffer (P). Additionally, it appears that despite exhaustive extraction at each step, several proteins repeatedly appear across fractions. As solubility is a robust indicator of protein biophysical state, we reason that the reason behind the various extraction patterns is the different biophysical affinities of individual protein populations for different GdnHCl ionic strengths.

### GdnHCl Extraction of *in situ* NuMat Preparation

To cross-examine the solubilization patterns seen in the GdnHCl SDS-PAGE, we probed for key NuMat proteins in GdnHCl extracted *in situ* NuMat using immunofluorescence. NuMat prepared from purified *Drosophila* nuclei is very sticky and does not lend well to confocal microscopy. We prepared *in situ* NuMat from intact uncrosslinked fly embryos using a slightly modified protocol (49, 50). This method also uses salt extraction and DNase treatment to remove chromatin and chromatin-associated proteins from uncrosslinked nuclei and hence replicates the biochemistry of NuMat in a microscopy friendly manner (50). We sequentially extracted the *in situ* NuMat with GdnHCl extraction buffers and found that the extracted embryo cage remains stable till 3M GdnHCl extraction and can be used to assess and further understand the GdnHCl extraction patterns of NuMat proteins.

We picked highly abundant nuclear proteins associated with different nuclear compartments and organizational processes that are also seen to be abundant in NuMat preps across species. These were heterochromatin protein 1 a (HP1a), polycomb (Pc)), megator (Tpr) and RNA Pol II subunit 215 (RNAP2)), Lamin Dm0 (Lam), fibrillarin (Fib), and GAGA-associated factor (GAF/Trl). We compare the embryo cages with intact nuclei (Nu), *in situ* NuMats (Nm), 1M-extracted (1M), 1M-2M-extracted (2M), and 1M-2M-3M-extracted (3M) (Fig. 1). Hp1 and Pc are largely chromatin-associated and remain only in a few foci upon NuMat preparation as the chromatin is removed by DNase I digestion. The NuMat-associated subpopulations of Hp1 and Pc are extracted mostly in low GdnHCl (1M/2M) extractions (Fig. 1, *A* and *B*). GAF is known to be a highly promiscuous chromatin-organizing pioneer transcription factor (57, 58). While a lot of GAF is removed with chromatin, the tight foci are retained in NuMat and extracted in a gradual manner (Fig. 1*C*). Lamin is the principal structural component of nuclear lamina (NL). Lamin is also known to have its subpopulations involved in many subnuclear processes off the scaffold, in the nuclear interior (59, 60). Similarly, a subpopulation of Megator/Tpr is the key structural component of the nucleoplasmic basket of nuclear pore complex (NPC) while much of it also localizes to chromatin and spindle matrix (61, 62). In NuMat, megator and lamin have primarily peripheral localization and they hold their place even after 3M GdnHCl extraction (Fig 1, *D* and *G*). The nucleolar foci of fibrillarin remain intact with NuMat preparation and 1M GdnHCl extraction but their morphology starts to change as we go to higher concentrations (Fig. 1*E*). The main transcription subunit RNAP2/Polr2A was probed with an antibody against its c-terminal domain with serine 5 phosphorylation, which is more associated with transcription initiation although it is seen in several combinatorial subpopulations of Polr2A subunit (63, 64). The CTDpS5 containing Polr2A moieties have pan nuclear foci that remain tightly bound through the GdnHCl extractions (Fig. 1*F*).

Thus, we observe that various NuMat proteins show highly divergent extraction patterns. Hp1a and Pc are easily solubilized while RNAP2, Tpr, fibrillarin, Trl, and lamin have significant difficultly solubilized subpopulations. Fibrillarin, lamin, and Trl show some gradual extraction patterns, indicating that they might have multiple subpopulations. Fibrillarin and HP1 are highly segregated scaffold proteins, building compact liquid-like environments in their respective compartments by multivalent interactions and association with nucleic acids (65, 66). However, they have very different behaviors as HP1 is extracted with the NuMat preparation from nuclei and in low-concentration GdnHCl washes while subpopulations of fibrillarin get extracted at all GdnHCl concentrations. In general, it is worth looking at how these differentially extracted subpopulations of proteins form complex compartments, that get differentially extracted in such a starkly distinct manner (as seen in Fig. 1, *A* and *E*). While much is known about the roles of both GAF and RNAP2 in a regulatory and mechanistic sense, their dynamics in the subnuclear space needs further exploration. However, both proteins appear to constitute a dense distribution of foci across the nuclear interior (Fig. 1, *C* and *F*). The differential extraction data suggest that while GAF is a protein with many extraction modalities, RNAP2 remains unextracted (supplemental Fig. S8).

NuMat reflects the well-established mosaic compartmentalized nature of eukaryotic nuclei. The observations with *in situ* NuMat elaborate that different proteins might be showing particular extraction patterns due to their biophysical status and their engagement with various subnuclear compartments. This also establishes *in situ* NuMat as a good platform for further biochemical and genetic investigations of solubility patterns of individual proteins.

### Proteomics of GdnHCl Fractions of NuMat

We observe very divergent solubilization patterns of NuMat proteins in the SDS-PAGE gel profile and upon sequential GdnHCl extraction of *in situ* NuMat. This presents a limited and complex picture of the nature of component organization within NuMat. To understand the GdnHCl solubility patterns of NuMat proteins *en masse*, we performed proteomics on the GdnHCl extraction fractions of the NuMat, with one more extraction step of 8M (1M, 2M, 3M, 4M, 8M, and P) (experimental schematic in Fig. 2*A*, parameters and file details in Supplemental Table S1). Summing up all the proteins across different fractions, 908 proteins were identified (Supplemental Table S2 sheet 1). Pairwise comparison of the protein lists of fractions shows that there is a significantly higher overlap between the proteins that are extracted at proximal concentrations (*e.g.*, 1M and 2M) and a smaller overlap at distal concentrations (*e.g.*, 1M and 8M), implying that the extraction is gradual (Fig. 2*B* and Supplemental Table S2 sheet 2). We consider the proteins solubilized with low ionic concentration GdnHCl buffers (1M-2M) as easily solubilized and the proteins only solubilized with 8M GdnHCl or not solubilized even by 8M GdnHCl (8M-P) as difficultly solubilized.

The sets 1M-2M and 8M-P have large numbers of unique proteins while the set 3M-4M does not, *e.g.*, 1M-2M: 1M (55), 2M (46), 1M Ո 2M (46), and 8M-P: 8M (153), P (57) 8M Ո P (88) (Supplemental Fig. S5 and Supplemental Table S3). The PCA of the quantitative proteome profiles of all replicates of all six fractions shows that the proteome intensity profile of the replicates of individual fractions are similar, and the fractions are distinct from each other. Also, a bigger divergence is seen between easily solubilized and difficultly solubilized proteins (Fig. 2*C*, PCA input data in Supplemental Table S4 sheet 1, summarized for visual inspection in Supplemental Table S4 sheet 2). We compared the quantitative profiles of the GdnHCl fractions by doing clustering analysis on differentially enriched proteins (*p* < 0.001) with a high fold change (>25log_2_), red indicating a heavily enriched protein and blue indicating a heavily depleted protein (Fig. 2*D*). The heavily differentially enriched proteins make up ∼16.5% of the total proteins detected (150 out of 908) (Supplemental Table S5 sheet 1). Two major clusters of heavily enriched proteins emerge in this analysis, which represent classes of proteins that are extremely easy to solubilize (1M-2M) and those that are extremely difficult to solubilize (8M-P) (Fig. 2*C*). We reason that the differential solubility of these proteins is because of their broad biophysical affinity to a particular GdnHCl concentration. This could be due to biophysical properties intrinsic to their amino acid sequence or due to extrinsic factors like macromolecular interactions or posttranslational modifications—dictating the manner of their spatial organization or packing in NuMat. However, we observe that the protein sets belonging to different extraction fractions do not show any significant enrichment in biophysical properties intrinsic to the amino acid sequences, *e.g.*, molecular weight, isoelectric point, intrinsic disorder, and percentage nonpolar amino acids (Supplemental Fig. S6 and Supplemental Table S6).

Since the extractions are finite (Supplemental Fig. S3), and yet the same proteins are extracted in multiple fractions, we surmise that there are multiple biophysical subpopulations of the same protein, leading to their differential extraction or solubilization. This becomes evident looking at ubiquitously solubilized proteins. Comparing qualitative profiles of all fractions together, we observe that 132 proteins are common in all fractions (Supplemental Fig. S5). In terms of LFQ too, a significant number of proteins (∼11.3%, 103 out of 908) are ubiquitous or display very low fold change (<2), which is statistically insignificant (*p* > 0.05) (Supplemental Table S5 sheet 2). In addition, a large number of proteins show extraction enrichment in different extraction concentration ranges (say 1M and 8M). Such extraction patterns could be considered spurious if they were seen to happen with one or two outliers. However, a large number of proteins show bimodal or multimodal extractions, which implies that the individual proteins have multiple biophysical subpopulations due to modifications or interactions with other moieties.

**Supplementary Figure S7:**
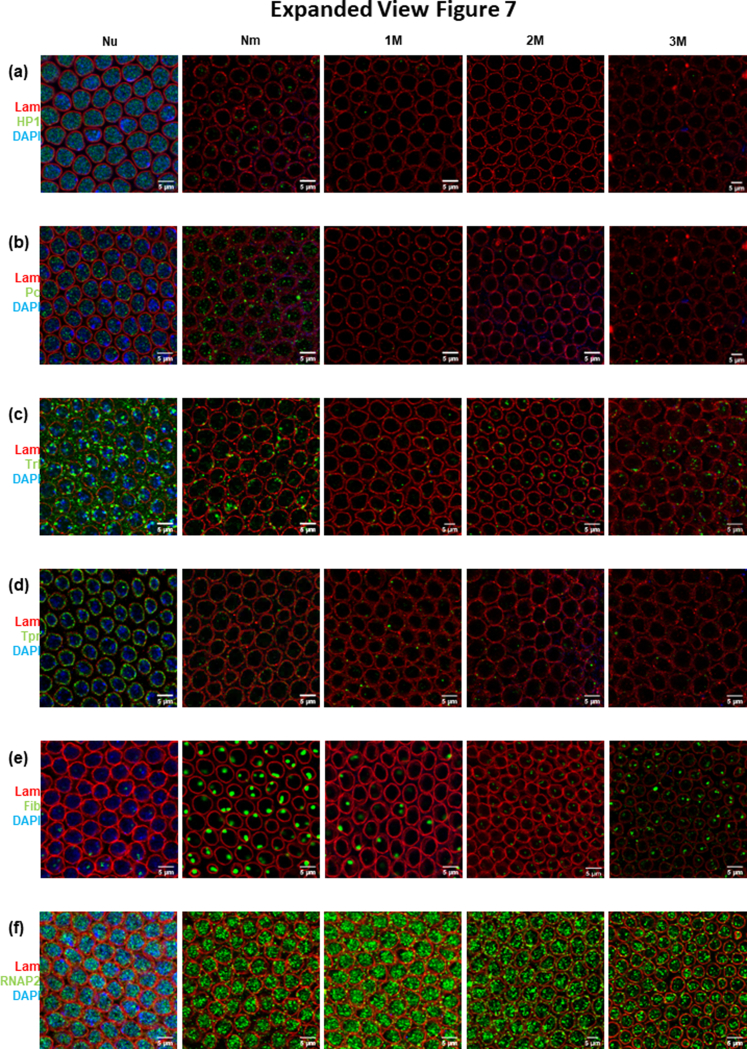
**Nuclei retain their shape through GdnHCl extractions.** Each *in situ* NuMat preparation at each extraction step (Nu for whole nuclei, Nm for in situ NuMat, 1M, 2M, and 3M) was stained with DAPI and immunostained for Lamin Dm0 alongside the target protein ((*A*) HP1 (*B*) Pc (*C*) Trl (*D*) Tpr/Megator (*E*) Fib (*F*) RNAP2. The figures show the sum projection of a few slices from the sheet of nuclei images at 100× magnification and 3× zoom, the Lamin staining showing that the nuclear morphology is intact through the sequential extraction, and DAPI staining showing that the chromatin has properly been removed during *in situ* NuMat preparation. Scale bar 5 μm in all images.

**Supplementary Figure S8:**
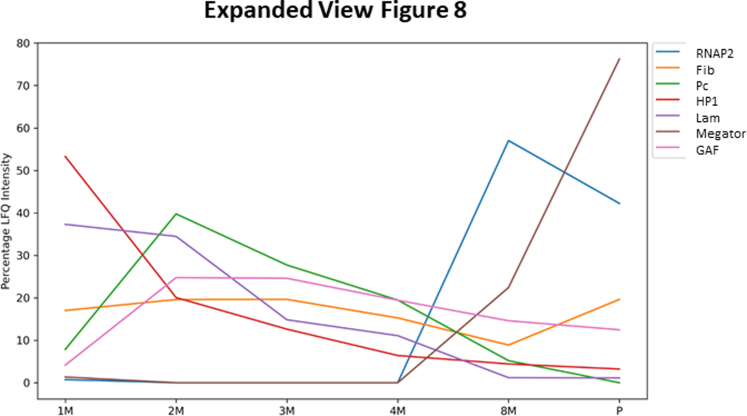
**LFQ-intensity-based extraction patterns of key NuMat proteins.** Shown here is the extraction pattern of NuMat protein examined through immunofluorescence and confocal microscopy of *in situ* Nuclear Matrix as line diagrams in terms of percentage extraction at each extraction step, X-axis showing the extraction steps (1M to P) and Y-axis showing percentage LFQ-intensity (0-100). Color codes for individual proteins are shown at the side of the graph. We observe that RNAP2 and Megator show no solubilization in the first three steps, HP1 and Pc are solubilized easily while GAF and Fib are ubiquitously-solubilized. Lamin shows easy solubilization here and ubiquitously solubilization in *in situ* Nuclear Matrix.

Depending on the environment of the protein in various nuclear subcompartments, the organization of the biophysical subspecies of a protein in the subnuclear space extrinsically shifts the protein’s propensity toward being easily solubilized, difficultly solubilized or being subdivided into different subpopulations with different solubilities *i.e.*, ubiquitously solubilized. Thus, sequential extraction with increasing GdnHCl concentrations clears off loosely bound and accessible material, leaving behind tightly packaged proteins. This is clearly observed in the case of relatively well-characterized complexes like NL and NPC (67, 68). In the case of the lamina, the core structural protein lamin is present in all fractions but, as seen in the microscopy images (Fig 1 and Supplemental Fig. S7), the retention of the peripheral lamin mesh is very prominent. This is possibly due to multiple subpopulations of lamin, some engaged in forming the peripheral mesh, others interacting with various complexes across the nuclear space (59, 60). Other NL proteins Man1 and LBR have previously been observed to be unsolubilized by 4M urea (69) and 8M urea (70). We observe that Man1 is indeed difficult to solubilize (exclusive to 8M). In contrast, Hp1 and LBR are easier to solubilize (depleted in 8M-P), as they anchor membrane and chromatin onto the lamina (71). In the case of the NPC, the members of the tightly packed pore (membrane anchors (NDC1 and Gp210), inner ring complex (Nup93, Nup205, and Nup154), outer ring complex (Nup107, Nup133, Nup160, Nup37, Nup44a, and Nup75, but not Nup98–96 and sec13), and filamentous mesh on the nuclear side (Tpr or Megator) and cytoplasmic sides (Gle1, mbo, and Nup358) are very difficult to solubilize. In contrast, the pore interior proteins (Nup62, Nup58, and Nup54) are readily solubilized and are strongly depleted in 8 M/P. Thus, the differential extraction patterns of NuMat proteins indicate a multilayered organization of proteins in nonchromatin space, broadly categorized into easily solubilized/loosely bound components and difficultly solubilized/tightly packed scaffold components.

### Renaturation of GdnHCl Solubilized NuMat Proteins

**Supplementary Figure S9:**
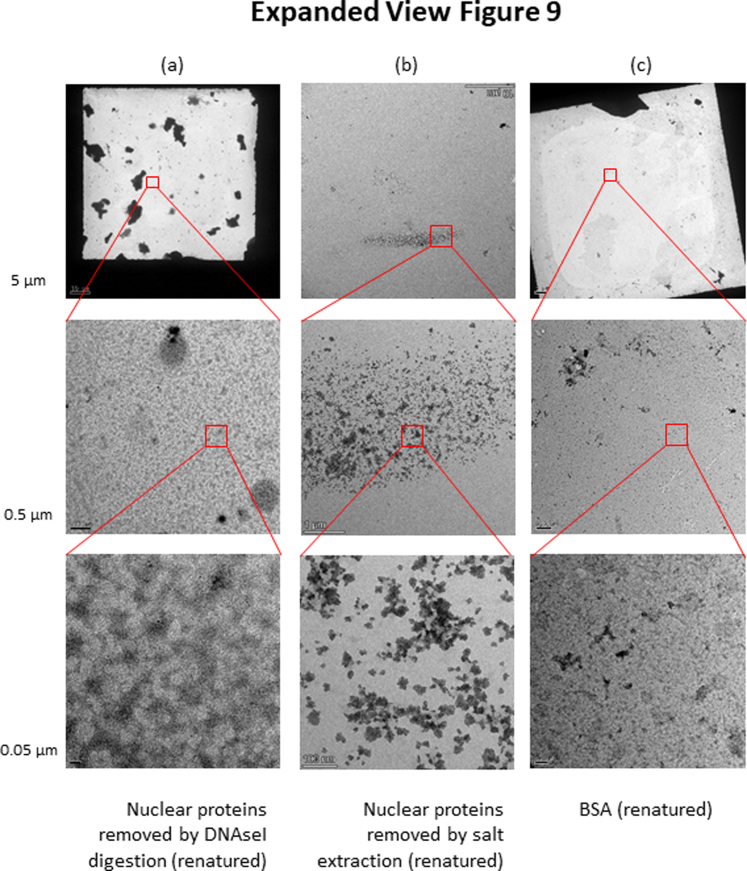
**Non-NuMat nuclear proteins don’t self-assemble to form fibers.***A*, DNase I extracted chromatin-associated proteins, (*B*) Salt extracted nuclear proteins (*C*) homogeneous protein solution with a single member, BSA. For all experiments, pictures were taken at three different magnifications (scales of 5 μm, 0.5 μm, and 0.05 μm). In the case of extreme magnification from 0.5 μm scale to 50 nm scale, the magnified area is outlined in a red circle.

**Supplementary Figure S10:**
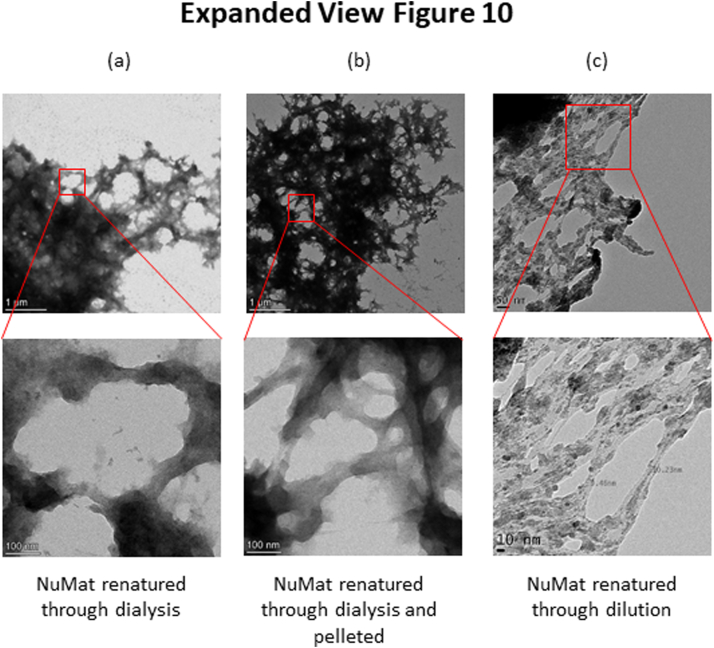
**Morphology of renatured NuMat fibers upon physical perturbations.***A,* NuMat renaturation fibers formed by dialysis and the grid dipped in renaturate (*B*) NuMat renaturation fibers formed by dialysis were spun down at 21000*g*, resuspended in 10 μL water and spotted on the grid (*C*) NuMat renaturation fibers formed by serial dilution (8M->4M->2M->1M->0.5M) and 10 μL renaturate spotted on the grid.

We observe that the proteins known to form filamentous meshwork assemblies through canonical polymerization and depolymerization, for example, actin, myosin, lamin, and so on, are easily solubilized. Instead, the difficultly solubilized fraction retains several key organizational and functional factors (Tpr, Top1/2, SMC2/4, RpII140/Polr2D, RpII215/Polr2A, Prp8, and so on.). Recent developments in the study of biomolecular condensates have shown that homotypic or heterotypic self-association among nuclear proteins is crucial for the hierarchical organization and membrane-less compartment formation in nuclei (72). While these studies provide valuable tools to look at self-association among soluble proteins, similar studies are difficult to do for insoluble endogenous proteins (73). To assess if NuMat proteins have any propensity for self-assembled ultrastructure formation, we dissolved the whole NuMat in 8M GdnHCl solution and cleared the undissolved fraction of debris (experimental schematic in Fig. 3*A*). The supernatant does not have any complex structure (Fig. 3*B*). Upon GdnHCl removal through dialysis, imaging the resultant sample with a transmission electron microscope shows the formation of networks of fibers (Fig. 3*C*). This shows that NuMat proteins can self-assemble into higher-order ultrastructures. The phenomenon of fiber formation by self-association is not observed in the renaturation of nuclear proteins extracted during NuMat preparation at the DNase I digestion step, and salt extraction step, or bovine serum albumin, despite using similar total protein concentration in the suspension (supplemental Fig. S9). This implies that not all sets of proteins can assemble through homotypic or heterotypic self-association. These fibers also form if GdnHCl concentration is reduced through dilution, also diluting the protein content drastically and therefore, are not dependent on protein concentrations (Supplemental Fig. S9). The fibers do not collapse when centrifuged at 21000*g* (supplemental Fig. S10*B*). Treatment of renatured NuMat with RNAse leads to the absence of fibers (Fig. 3*D*). We also prepared NuMat with RNAse treatment alongside DNase treatment. The renaturation of proteins from this prep also leads to many proteins becoming insoluble but they do not form fibers (Fig. 3*E*), implying that nuclear RNA is an important driver of the ultrastructure forming self-assembly of NuMat proteins.

Thus, NuMat consists of proteins that have the propensity to be assembled in this manner (Supplemental Fig. S10, *A*–*C*). To further understand the relationship of protein solubility with the propensity for reassembly through renaturation, we removed GdnHCl from the extraction fractions through dialysis. The presence of renatured fibers was seen only in the 8M fraction (Fig. 4). Thus, the RNA-dependent homotypic or heterotypic self-association of difficultly solubilized NuMat proteins results in the formation of these fibers. Furthermore, these findings would apply to the completely insoluble fraction (P) (Fig. 2) obtained through sequential extraction, left out in the renaturation experiments because of its insolubility.

### Proteomics of Renatured Fraction

**Supplementary Figure S11:**
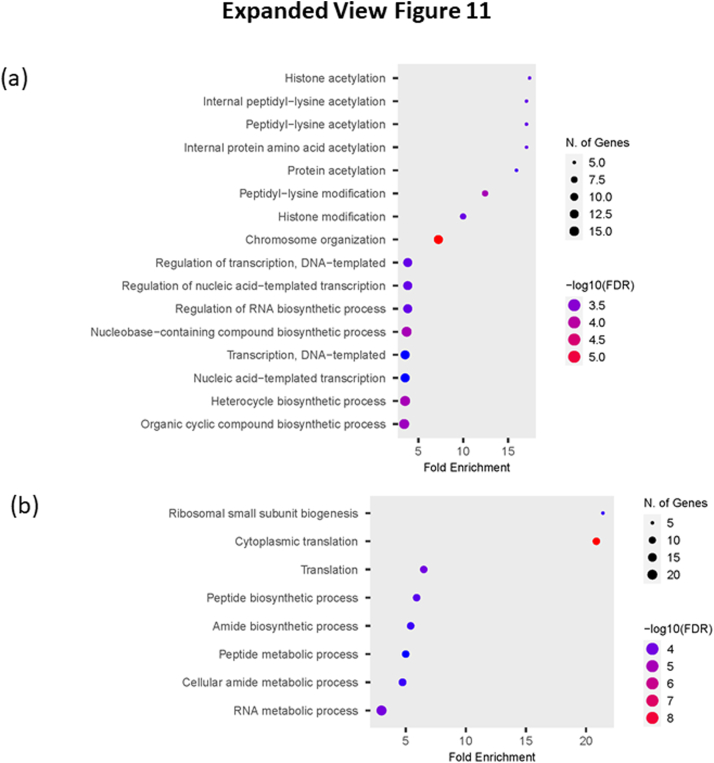
**Gene ontology analysis of two major subsets of core renaturation factors, shown here are dot plots where Y-axis shows various biological processes enriched, X-axis shows fold change, the dot size shows absolute protein count for a pathway and the dot color represents the p-value of enrichment (*red* means more significant).***A*, Gene Ontology analysis of core renaturation proteins overlapping with proteins unique to 8M (*B*) Gene ontology analysis of core renaturation proteins overlapping with 8M proteins that are common to all fractions. Plotted with ShinyGO.

To understand further the nature of the NuMat proteins renaturing through self-association, we pelleted down the renatured fibers from the dialyzed suspension and performed proteomics on them (Supplemental Table S7 sheet 1). PCA of this dataset with extraction fractions and nonfiber forming fractions (DNase I digestion fraction and salt extraction fraction) shows that this set of proteins is very distinct from nonfiber forming fractions (Fig. 5*A*). Comparison of fiber-forming renaturation fraction with nonfiber forming fractions yields a cluster of 198 proteins—with statistically significant and high fold change enrichment in renaturation fraction (Fig. 5, *B* and *C*) (Supplemental Table S7 sheet 2), which we consider as core renaturation components. These factors are enriched in GO terms related to histone modification, transcription, and chromosome organization (Fig. 5*D* and Supplemental Table S7 sheet 2). When comparing this cluster with individual extraction fractions, we find that the largest overlap is with the 8M fraction, agreeing with the observations from fraction-wise renaturation (Figs 4*E*, 5*E*, and Supplemental Table S7, sheet 3). Here, we see that the renaturation overlap proteins only present in 8M and absent in other extraction fractions are GO enriched with terms related to transcription regulation, and chromosome organization. In contrast, renaturation overlap proteins common in all fractions are enriched in terms related to translation and ribosomal biogenesis (Supplemental Fig. S11, *A* and *B* and Supplemental Table S8 sheet 2). A breakdown of these proteins according to FlyBase annotations has been shown in suppl. file 2. Based on the extraction fraction-specific renaturation shown in Figure 4*E*, the ubiquitously solubilized proteins are also contributing to renaturation through difficultly solubilized subpopulations. They could be switching between their various biophysical states, which would provide the dynamic component to the self-assembled scaffolds.

The abundance of RNA-binding proteins among the total renaturing factors reasserts the importance of RNA in bringing large complexes together and setting a structural framework. This explains why the reassembly collapses when RNA is eliminated (Fig. 3*D*).

### Extraction Patterns of the Renatured Proteins

To understand the nature of core renaturation factors, we look into their solubility through their extraction patterns. Firstly, we group all the proteins based on their extraction patterns by applying k-means clustering on the DEP-enrichment scores of each protein in different fractions. Figure 6*A* depicts the DEP-enrichment scores of proteins in all the fractions across the 13 clusters. We observe the emergence of three prominent groups (Fig. 6, *B*–*D*) with specific extraction patterns that show significant overlap with renaturation factors. First is the set of difficultly solubilized proteins which are extracted at 8M and also are retained in the pellet (Fig. 6*B*). Among the 60 proteins which are extracted in this pattern, 30 are renatured. Next, 63 proteins are only extracted at 8M, and 14 of them are renatured. Also, a major set of renatured proteins are among the ubiquitously extracted proteins (49 out of 118 proteins) (Fig. 6*D*). GdnHCl coextraction does not indicate collective behavior in multiprotein complexes. Instead, some members of individual complexes show easy-to-solubilize nature and others are difficultly solubilized, indicating a differential role in the recruitment and positioning of the complexes with spatiotemporal variation. The 8M-P overlap pattern of difficultly solubilized proteins is enriched in architectural and organizational factors. For example, structural maintenance of chromosomes (SMC) complex components SMC1, SMC2, SMC3, SMC4/glu, SA1/Scc3, Cap-G, and Cap-D2 are part of extraction pattern 1 (Fig. 6*B*). While the core components of condensin complexes, that is, SMC2 and SMC4/glu (present in both 8M and P) are part of renaturation factors, Cap-D2 and Cap-G (present mostly in 8M; Fig. 6*C*) are not. SMC2-4 proteins have also been observed to be abundant in mitotic chromosomal scaffold, a biochemical preparation analogous to that of NuMat (33, 74). This scaffold seems to form through geometric segregation of cohesin and condensin complexes (75) which possibly leads to their assembly into insoluble ultrastructures that form a basis for hierarchical organization.

## Discussion

Several investigations on the whole-cell proteome have identified highly insoluble proteins in mammalian cells. A small fraction of proteins change their solubility status upon binding ATP and RNA, and are implicated in the formation of condensate scaffolds through posttranslational modifications (PTMs) and signaling (11, 12). However, a large number of insoluble proteins do not change their solubility status. Using stepwise extraction with different concentrations of GdnHCl provides a way to further dissect and analyze the difficultly solubilized proteome (2, 76)—which we do here for the nuclear nonchromatin insoluble fraction, that is, NuMat. NuMat is the insoluble component of the eukaryotic cell nucleus, resistant to dissolution upon DNase I and salt-detergent treatment, and retains the spatial organization of the nucleus (19). In the absence of characterized subfractions of NuMat, we establish GdnHCl-based stepwise extraction as a method to gradually and exhaustively solubilize NuMat proteins. Given the recent emergence of observations showing the organization of semisolid and solid condensates in the subnuclear space (77), the solubility profile of NuMat can be utilized to further understand the structural basis of nuclear compartmentalization.

While both GdnHCl and urea are chaotropes able to unfold proteins, GdnHCl can shield ionic interactions due to its charge (78). Therefore, the observation that different concentrations of GdnHCl but not of urea can solubilize proteins from NuMat indicates that the association of the solubilized protein with the undissolved substratum is ionic (Supplemental Fig. S2, *A* and *B*). To check if the partial solubilization of NuMat is due to the saturation of the extraction buffer, we analyze the pattern of protein solubilization with multiple sequential extractions of NuMat at the same concentration of GdnHCl. We observe that all GdnHCl extraction concentrations solubilize a finite amount of protein in a finite amount of time, with negligible proteinaceous material leeching upon further extraction (Supplemental Fig. S3). Thus, individual extraction fractions performed in a sequential manner can be considered distinct from each other (Fig. 2*C*). The reproducible proteomic profile of GdnHCl-based sequential extraction fractions of NuMat leads us to reason that the sequential solubilization of NuMat proteins is not random and is due to their biophysical propensity to be solubilized at a particular concentration and detach from the undissolved substratum in NuMat (Fig. 2*C*). This could either be due to biophysical properties intrinsic to the proteins’ amino acid sequence or the creation of various biophysical subspecies through PTMs and heterotypic or homotypic macromolecular interactions. Upon analysis of the proteomic profiles of GdnHCl extractions fractions, we find that the broad biophysical properties of proteins intrinsic to their amino acid sequences that could affect the solubilization behavior pattern of proteins, or where an extraction bias would be reflected, are uniformly distributed across the GdnHCl extraction fractions (Supplemental Fig. S6). Therefore, a protein’s differential strength of association with the undissolved substratum depends on PTMs and macromolecular interactions. The scaffold formation ability of proteins through polymerization or phase separation and their spatial organization has been observed to be modulated by PTMs in the case of Hp1 and lamin, among others (60, 79). Further exploration of the PTM landscape in the differentially solubilized subpopulations will add to the power of GdnHCl-based extractions.

We check the solubilization patterns for components of NL and NPC as the spatial organization of these nuclear substructures is relatively well characterized. We observe that components of NL and NPC have multilayered organization. The tightly packed scaffolds that are difficultly solubilized are decorated with loosely bound, easily solubilized material. As inferred from staining for lamin in *in situ* NuMat, the low-concentration extractions do not disrupt the nuclear shape, wherein a similar trend of extraction at 1M, 2M, and 3M GdnHCl is seen for key NuMat proteins (Fig. 1). Additionally, we see that while GAF and RNAP2 are organized in pan-nuclear foci, other NuMat proteins are specific to subnuclear domains, which agrees with the current understanding of nuclear subcompartmentalization (80). This aligns with the idea that the distribution of proteins in NuMat is a snapshot of their distribution in nuclei (81).

In light of these outcomes, let us consider the three possibilities for the organization of components in NuMat that we delineated in the introduction. NuMat does not behave like a canonical polymeric activity-driven fibrous structure as the proteins with the fiber-forming activity are solubilized easily (I). NuMat also does not behave like a random aggregate in which proteins would have different solubility profiles in different NuMat preps (III). Instead, what we find is that we are capturing and dissecting the spatial organization of various nuclear substructures together (II). It should be noted that several recent studies have implicated RNA-protein interactions and subsequent condensate formation in the self-assembly of nuclear subcompartment scaffolds. These scaffolds have been seen to behave in liquid, solid, and semisolid manners and cause the enrichment of different subspecies of subcompartment-associated markers (72, 73). Additionally, the existence of higher-order filament-like substructures in such condensates has been detected using Cryo-ET (9).

Therefore, we explored the possibility of the *in vitro* reassembly of NuMat components. To check if NuMat proteins can independently self-assemble, we dismantle the NuMat in 8M GdnHCl and ask if it can renature and form ultrastructural features if the chaotrope is removed by dialysis. As shown in Figure 3, NuMat proteins solubilized by 8M GdnHCl show a unique propensity to self-assemble into fibers. No reassembly is seen upon renaturing protein suspension of similar concentration consisting of chromatin-associated proteins extracted in DNase I/salt fraction (obtained during NuMat preparation) (Supplemental Fig. S10, *A* and *B*). Upon comparing the proteome of the renatured NuMat fibers to the proteome of DNase I and salt extraction fractions, a cluster of proteins enriched in renatured fibers emerges (Fig. 5, *B* and *C*). We reason that these proteins are highly likely to be causative to fiber formation through heterotypic or homotypic association among themselves, mediated by RNA (Fig. 3, *C* and *D*). This further emphasizes the role of RNA in the architecture of the nuclear interior as claimed in old electron microscopic studies of NuMat fibers (36) and recent observation of key NuMat proteins (MATR3, NUMA, and SAF-A) forming a subnuclear RNP network segregated from chromatin (82).

The renaturation of extraction fractions combined with the analysis of the overlap of the core renaturation components with extraction fractions shows that the difficult-solubilized subpopulations of endogenous nuclear proteins readily renature (Figs 4*E*, and 5*E*). The component of renaturation factors that is unique to the 8M extraction fraction is enriched in GO terms related to chromosome organization, histone modifiers, and control of transcription (Supplemental Fig. S11*A*). In contrast, the more ubiquitously solubilized protein component is enriched in GO terms associated with ribosomal subunit precursors and splicing (Supplemental Fig. S11*B*), showing functional divergence between the two subtypes. A group of proteins observed to be enriched in 8M-P that also are prominent in renaturation factors, are the members of cohesion and condensin complexes (Fig. 6*E*). Self-association among SMC2-4 dimer units through long DNA molecules has been observed to form DNA-condensin condensates (Ryu *et al*., 2021). SMC2-4 dimers also have been seen to bind long RNA molecules, suggesting an alternate way for them to form insoluble condensates (83). Across different organisms, several of the difficultly solubilized epigenetic factors that also renature have been observed to be directly or indirectly associated with RNA, implicating these protein–RNA interactions in the organization of chromatin in the nucleus (84) like Set1 (85, 86), Uba1/2 (87) Cul4 (88), etc. We speculate that the difficultly solubilized proteins form domains with RNA acting as an indispensable building block of the pan-nuclear architectural framework.

The placement of a protein in such a hierarchical organizational framework can be a regulatory choice. Looking at previous work on NuMat targeting signals (NMTS) of various proteins in this context, studies have narrowed down NMTS patches on proteins like Beaf32 and Pho (YY1 homolog) (89, 90) among others (91). In the absence of the putative NMTS, the protein remains nuclear but changes its subnuclear compartment, also changing its solubility. In WT proteins, the solubility or scaffold association of architectural proteins could be regulated by PTMs, which, in the case of Beaf32, are found enriched directly on the NMTS patch (92). Our dataset can be a useful resource to examine such regulation through cell’s organizational control of protein solubility. However, we would like to add that further quantitative analysis of the dynamics of protein solubility across large-scale cellular and developmental processes is required for the present methodology to be effective. Our interpretation is limited by the fact that the datasets presented here represent the average of different cell states across a large 0 to 16-h developmental window.

In summary, we strip NuMat down sequentially, based on the differential solubility of its constituents, dissecting the spatial organization of proteins in the nonchromatin nuclear organization. The NuMat proteins form a multilayered organization through RNA-dependent mutual association. As we observe mosaicism in the protein components and the architectural importance of RNA in the reassembly of NuMat, we suggest that instead of canonical homopolymeric fibers, a noncanonical multicomponent assembly is involved in fibrous meshwork formation. Additionally, the solubility profile of this meshwork provides a proteome-wide biophysical tool to examine the dynamics of insoluble endogenous nuclear proteins and the liquid/solid/semi-solid scaffolds in various nuclear substructures they engage with. Whether the heterogeneity inherently embedded in such a structure brings in the necessary functional multiplicity remains to be explored. The *in situ* NuMat preparation combines *Drosophila* genetics and cell biology approaches will accelerate the exploration of key NuMat components outlined here in the ultrastructural context.

## Data Availability

The raw proteomics data are available *via* ProteomeXchange with the identifier PXD031296 (login: Username: reviewer_pxd031296@ebi.ac.uk Password: 7FmlIU1M). The filenames corresponding to biological replicates and their fractions have been provided in Supplemental Table S1.

## Supplemental data

This article contains supplemental data (48).

## Conflict of interest

The authors declare no competing interests.

## Supplementary Data

Supplementary file 2

Supplementary Table S1 PRIDE raw spectra file summary and MaxQuant parameters: This table has one sheet. Column A-G contain the summary of raw spectra filenames of files provided in PRIDE repository and their associated experimental specifications (for both sequential extraction and renaturation). Column K-L contain the parameters used for Maxquant analysis.

Supplementary Table S2 **Extraction fractions pairwise overlap:** This table contains two sheets. First sheet named “total numat qualitative” contains a qualitative summary of all protein identifications for sequential extraction that have at least 2 unique peptides and non-zero LFQ intensity in all three replicates of at least one extraction fraction. Second sheet named “fraction profile qualitative” contains lists of proteins in each fraction from “total numat qualitative” and pairwise overlap protein lists in terms of Uniprot IDs.

Supplementary Table S3 Extraction fractions all-to-all comparison: This table contains a single sheet with every possible overlap among the 6 fractions.

Supplementary Table S4 Quantitative summary: This table contains two sheets. First sheet named “PCA input” contains the MaxQuant output file where protein identification rows containing less than 2 unique peptides or zero LFQ values in all fractions and contaminants identified have been removed. Second sheet named “identifications qualitative” present a summary of total unique peptide and LFQ values for each fraction alongside protein IDs and percentage coverage.

Supplementary Table S5 Quantitative differential enrichment: This table contains two sheets. First sheet named “DEP_sig” contains protein ID and differential enrichment scores generated by DEP for proteins with at least 25log2 fold change across fractions and p-value below 0.001. Second sheet named “Proteins_leastvariation” contains the list of proteins which vary less than 2-fold with p-value above 0.05.

Supplementary Table S6 Biophysical properties: This table contains 18 sheets representing the proteomic profile of each replicate (3) of each fraction (6). Each sheet contains the molecular weight, isoelectric point, percent non polar amino acids, percent disorder for each protein associated with the fraction replicate.

Supplementary Table S7 Renaturation fraction analysis: This table contains three sheets. First sheet “ren quant summary” contains a quantitative summary of all identifications in renaturation fraction. Second sheet “CoreRenFactor_volcano” contains LFQ intensity based volcano plot comparisons of renaturation fraction with DNAse I digestion fraction and salt extraction fraction of Nuclear Matrix prep. Third sheet “CRF_extfraction” contains protein ID lists of all possible overlaps of Core Renaturation Fraction (CRF) with extraction fractions.

Supplementary Table S8 Renaturation GO analysis: This table contains two sheets, first one named “RenEnrichedFactorsGO” contains gene ontology analysis of Core Renaturation Fraction proteins together, and second one named “RenEnrichedSubsets” contains gene ontology analysis of ubiquitously-solubilized difficultly-solubilized subsets of Core Renaturation Fraction proteins.
