# Supplementary material for: Biochemical Deconstruction and Reconstruction of Nuclear Matrix Reveals the Layers of Nuclear Organization
Source: Mol Cell Proteomics. 2023 Oct 19;22(12):100671. doi: 10.1016/j.mcpro.2023.100671 (PMC10687341; doi:10.1016/j.mcpro.2023.100671)
Supplement: Supplementary file 2 [file mmc2.docx]

**Table: Broad categorization of core renaturation proteins**

| **Proteins** | **Function** |
| --- | --- |
| **Ubiquitously solubilized proteins** | |
| RpS8, RpS9, RpS11, RpS13, RpS14b, RpS16, RpS17, RpS18, RpS19a, RpS20, RpS27, RpL11, RpL18a, RpL21, RpL31, sta | Ribosomal subunit proteins (assembled in nucleolus) |
| eEF2, eIF4a | Translation regulation |
| IDH3a, Eno, Pgk, SkAP, Jafrac1 | Metabolic enzymes |
| Lark, lost, Pnn, HnRNP-K, x16, fl(2)d | Splicing regulator |
| Ibf1, Bap60, Bap170, Mi-2, Prod, Vgll4 (CG1737), Jigr1, Brd7-9, lolal, atms, Nap1, Crp, Hcf, Nab2, DMAP1, simj, HP5, ab | Chromatin or transcription associated |
| Pom121 (CG14712), Nup153, Nup35 | NPC, RNA export |
| CCT2, Nop17 (CG5792), 14-3-3 zeta, tsr | Others |
|  |  |
| **Difficultly solubilized proteins** | |
| Uba1, Uba2, Bre1, Cul4, CG7208 | Ubiquitination/SUMOylation |
| Nup153, Nup205, Nup160 | NPC, RNA export |
| Kdm4a, Gcn5, Br140, enok, Set1, Osa, Med17, MDB-R2, Sfmbt, PF1, IntS3, Nsl1, CG17385 | Chromatin or transcription associated |
| AspRS, RnrL, r | Metabolic enzymes |
| SMC2/4, Nipped-B, Rad50, PolD, SryD | Structural elements |
| HEATr1, eIF3e, | Ribosome assembly |
| xmas-2, Wdr33, Symplekin, Ge-1, Ntr2 | Transcript processing |
